# Analysis of the Spatial Organization of Molecules with Robust Statistics

**DOI:** 10.1371/journal.pone.0080914

**Published:** 2013-12-04

**Authors:** Thibault Lagache, Gabriel Lang, Nathalie Sauvonnet, Jean-Christophe Olivo-Marin

**Affiliations:** 1 Unité d’Analyse d’Images Quantitative, Institut Pasteur, Paris, France; 2 Unité de Recherche Associée 2582, Centre National de la Recherche Scientifique, Paris, France; 3 Unité Mixte de Recherche 518 Mathématiques et Informatique Appliquées, AgroParisTech and INRA, Paris, France; 4 Unité de Biologie des Interactions Cellulaires, Institut Pasteur, Paris, France; University of Birmingham, United Kingdom

## Abstract

One major question in molecular biology is whether the spatial distribution of observed molecules is random or organized in clusters. Indeed, this analysis gives information about molecules’ interactions and physical interplay with their environment. The standard tool for analyzing molecules’ distribution statistically is the Ripley’s K function, which tests spatial randomness through the computation of its critical quantiles. However, quantiles’ computation is very cumbersome, hindering its use. Here, we present an analytical expression of these quantiles, leading to a fast and robust statistical test, and we derive the characteristic clusters’ size from the maxima of the Ripley’s K function. Subsequently, we analyze the spatial organization of endocytic spots at the cell membrane and we report that clathrin spots are randomly distributed while clathrin-independent spots are organized in clusters with a radius of 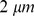, which suggests distinct physical mechanisms and cellular functions for each pathway.

## Introduction

Spatial organization of objects is essential in many scientific areas because it provides information about objects’ interactions and their interplay with their environment. Objects’ organization can be studied at different scales, ranging from country size in epidemiology [1] to atomic structures in physics [2]. For example, the study of the distribution of leukaemia cases in Britain between 1966 and 1983 in epidemiology revealed some geographical aggregation that may be related to environmental factors [3]. In ecology, the analysis of spatial patterns across ten years in an aspen-white-pine forest [4] showed that tree distribution tended toward greater clumping than that expected from random mortality, which is due to the clonal nature of aspen. At molecular scale, the quantitative analysis of gold particle distribution in electron microscopy helped to analyze the three-dimensional distribution of pyramidal neurons and the related neural circuits [5]. It also gave hints about the distribution of Ras proteins at the plasma membrane [6], [7] and the related organization of specialized micro-domains such as lipid rafts. Similarly, the analysis of the spatial distribution of fluorescent markers attached to proteins of interest in confocal microscopy shed light on underlying mechanisms of various cellular processes, such as signaling at immunological synapses [8], and can be used to measure cellular phenotype changes in different conditions, such as during pathogen infection [9].

**Figure 1:**
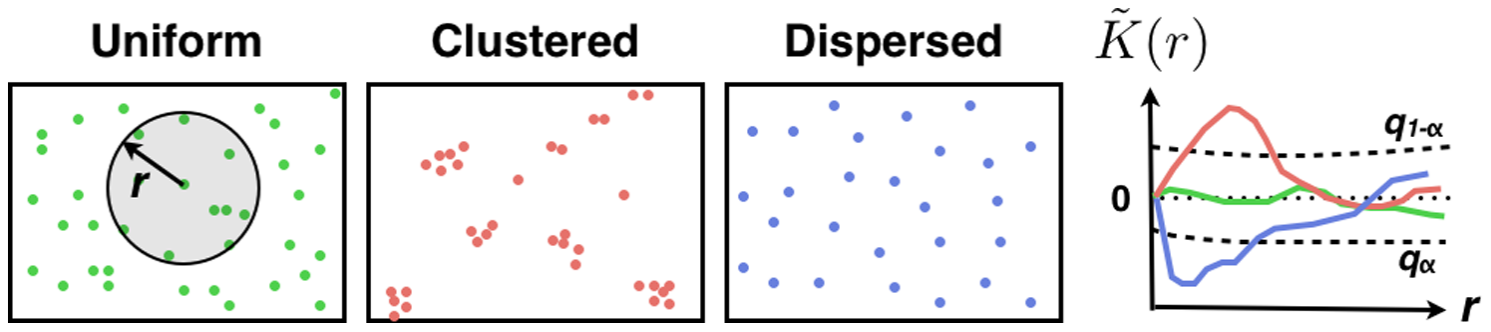
Analyzing spatial point patterns with Ripley’s K function. The normalized and centered Ripley’s K function 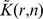 is proportional to the number of pairs of points that are closer than 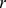 in 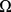. Deviations of 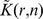 from 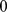 (CSR) in clustering 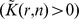 or dispersion 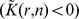 conditions have to be compared with objective level of significance that are quantiles 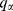 of 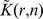 at level 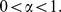

In all spatial organization studies, objects (disease cases, trees, molecules …) are represented as points in a delimited field of view (country, forest, cell …) and quantitative methods are used to extract features about spatial point distributions. Classical methods are either area-based or distance-based. In the first case, the points’ pattern is characterized through its first-order properties such as the spatial variation of its points’ density, which is often estimated with patches or kernel methods [10], whereas in the second case, distance-based methods rely on second-order properties of the points’ pattern such as inter-point distances, and a major milestone was established by Clark and Evans (1954) who introduced statistics based on the distance of points to their nearest neighbors. An essential piece of information is given by the deviation of points’ distribution from complete spatial randomness (CSR) and the concomitant detection of specific patterns such as point clusters (Figure 1). Thus, the two major goals when building a quantitative method are: **1)** assess statistically whether observed specific patterns such as clusters are not due to chance, that is to say points are not randomly distributed in the field of view, and **2)** determine the characteristics of the observed patterns such as the clusters’ size. While the first goal is often achieved by the computation of the critical quantiles of the statistics used under CSR, the second one mainly involves fitting to parametric models.

However, these classical methods are plagued with some disadvantages: area-based methods cannot account globally for objects’ interactions, and nearest-neighbors methods do not describe objects’ interactions at several scales. To answer these problems, a great advance was made by Ripley in 1977 [11] who introduced the distance-based K function which describes the spatial organization of any point process quantitatively at several distance scales by taking into account all neighbors rather than only the nearest. Yet, Ripley’s K function still presents some problems. First, there is no analytical formula that links the critical quantiles of the K function to the number of points and the geometry of the field of view. Consequently, the computation of the critical quantiles is based on intensive Monte-Carlo resampling, which induces an high computational load and requires an initial calibration for each field of view due to specific edge effects. Second, the quantification of pattern characteristics is also problematic because model parameters are currently extracted from fitting procedures involving a functional minimization, such as least square method; few efforts have been made to directly extract key spatial features such as the cluster radius or the minimal distance between dispersed points from the essential properties of the Ripley’s curve such as its extrema [12].

Here, we propose two major methodological improvements: **1)** give a closed-form expression of critical quantiles, and **2)** relate standard features such as cluster size to essential properties of the Ripley’s K function. Point 1 alievates the need for Monte-Carlo simulations and point 2 bypasses minimization procedures. Taken together, these two points give rise to a fast, robust and analytical method which is additionally implemented and freely available in Icy [13] (http://icy.bioimageanalysis.org).

Thereafter, we used this method to characterize the spatial organization of different endocytic pathways. Endocytosis is indeed a key mechanism for cell homeostasis whereby cells engulf signaling molecules and nutrients from the extra-cellular medium. The most frequent endocytic pathway is mediated by the clathrin protein that forms coats around specific receptors, leading to membrane invagination and molecules entry [14]–[17]. Many other important pathways do not rely however on clathrin, notably the internalization of interleukin 2 (IL-2) and its receptor (IL-2R) [18] during the cell mediated immunity [19], [20]. In both cases, the spatial organization of endocytic spots at the membrane still remains poorly characterized, while it might reflect localized cellular processes such as cell migration and signaling [21]. Here, we compare the spatial organization of clathrin-dependent and -independent endocytosis. We report that both pathways are regularly organized at small distance (for 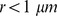). At larger distance scales, clathrin-independent pathways exhibit clusters with a radius of about 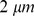 while clathrin-dependent putative endocytic sites are randomly distributed.

## Results

### Construction of the Test Statistic

We aim at constructing a statistic 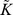 to test whether a points’ distribution is random or clustered by comparing its values with critical quantiles under CSR (Figure 1). A standard statistic is the Ripley’s K function whose standard expression at distance scale 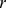, and for 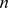 objects at position 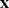 in a given field of view 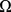, is
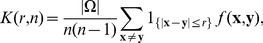
(1)where 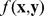 is a boundary correction term that prevents a bias in 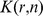 at larger values of 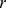 due to the finite size of 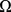. Indeed, some pairs of points closer than 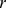 can fall outside the observation window 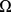, leading to an underestimation of 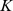. A widely used boundary correction is the Ripley’s correction 
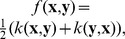
 where 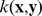 is inversely proportional to the proportion of the circle 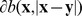 included in 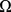: 
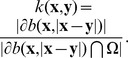
 With this boundary correction and under CSR, ([22], page 39)



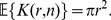
(2)The problem when using the standard Ripley’s K function (Eq.1) is that its mean and variance under CSR vary with distance scale 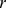, which complicates its quantitative interpretation. A partial answer has been proposed by Besag who introduced the centered 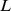 function [23] 
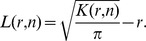
 However, 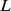 function is not normalized and we thus propose a new statistic with zero mean and unit variance that uses the analytical expression of 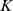 variance.

The computation of the variance 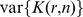 of 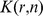 under CSR is made difficult by edge effects, but assuming that 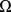 boundary is locally straight where it intersects 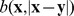, a closed-form expression of 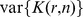 has been obtained by Ripley [22]:
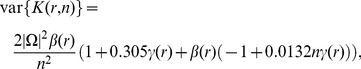
(3)where 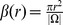 and 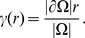


Using the closed-form expressions of the variance (Eq. 3), we introduce the normalized and centered statistics
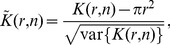
(4)whose significant deviations from 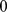 are characteristic of object clustering at length scale 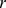 when 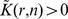 or dispersion for 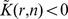 (Figure 1). To characterize these deviations statistically, we compute hereafter critical quantiles of 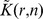 under CSR.

### Estimation of 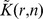 Critical Quantiles Under CSR

A first attempt of computing the critical quantiles of 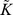 analytically was proposed by Lang and colleagues [24]. They decompose 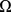 in independent sub-domains, and using the central limit theorem, they prove that for 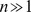, 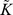 can be approximated by the standard normal law 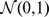 under CSR (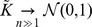). This is equivalent to approximate 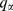, the quantile at level 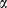 of 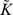, with 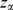, the quantile of the standard normal law 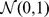: 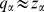.

This approximation does not hold for intermediate values of 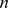 or for small distance scales 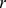 (see below), and we propose hereafter a general approximation of 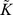 quantiles that is valid for a large range of 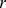 and 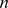 values. This is based on the standard Cornish-Fisher (CF) expansion which is given by [25]

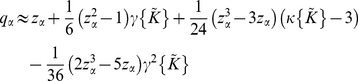
(5)where 
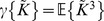
 and 
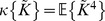
 are respectively the skewness and the kurtosis of 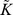. It can be deduced from Eq.5 that the CF expansion generalizes the central limit theorem which states that 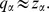


At this point, we still face a problem because we do not have expressions for the skewness and the kurtosis of 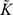, whose expressions are made difficult because of boundary effects. After long and mathematically involved computations which are detailed in File S1, the expressions of the skewness and the kurtosis of 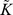 are given by
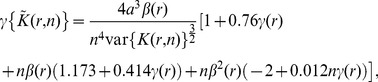
(6)and
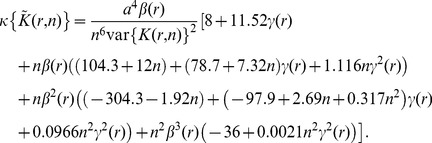
(7)with 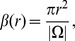 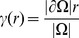 and 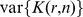 is given by Eq. 3.

At this point, we can make three comments: **1-** Setting apart the assumption that the boundary can be treated locally as a straight line, formulas for skewness and the kurtosis (Eq. 6–7) are exact. **2-** Reintroducing the approximations of variance, skewness and kurtosis (Eq. 3, 6 and 7) in the CF expansion (Eq. 5), we find that 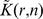 is asymptotically normal: 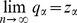 in agreement with [24]. **3-** In many applications, 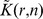 can be evaluated on 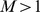 fields of view and it is then interesting to use the mean statistics
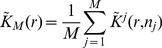
(8)where 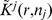 is evaluated on the 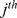 field of view. The CF expansion of 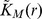 quantiles then requires the computation of the skewness and the kurtosis of 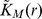, which is detailed in File S1, section V.

### Assessing the Specificity of our Statistical Test on Synthetic Data

To test the accuracy of the obtained CF approximation of 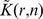 quantiles (Eq. 5), we tested it against intensive Monte-Carlo resampling in a given field of view. In addition, we also compared the *true* quantiles obtained with simulations with the standard normal approximation.

To ensure the convergence of the Monte-Carlo method, we performed 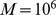 simulations where we drew uniformly 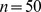 points in a 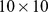 square 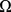. We then computed the corresponding Ripley’s K function 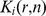 (Eq. 1), for 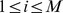 and 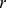 varying from 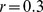 to 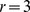. For each 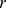, we computed the empirical variance 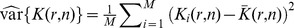 where 
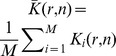
 is the empirical mean tending to 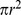 for 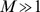, and we then obtained
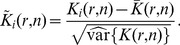
(9)


The empirical quantile 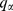 of 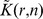, for 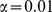 or 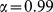 was then computed by sorting the 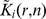 and choosing 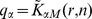 with the floor function of 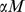.

**Figure 2:**
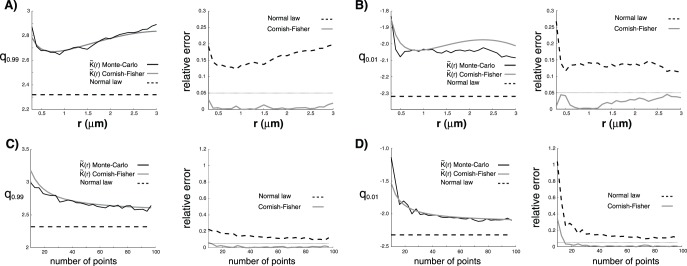
Test of CF expansion against Monte-Carlo simulations. A)-Left: The CF expansion (Eq. (5), grey line) of the quantile 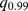 of 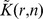 is tested against Monte-Carlo simulations (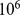 simulations, solid black line) in a 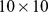 square 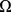 for 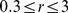. The number of points is set at 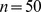. The quantile 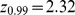 of the standard normal law 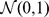 is also represented (black dotted line). A)-Right: Relative errors of CF expansion (grey line) and 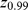 (black dotted line) to Monte-Carlo simulations. The 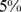 level is represented with a black dotted line. B) Idem to A) for the first percentile 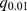 of 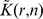 instead of the last one 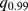. C)-Left: The CF expansion (Eq. (5), grey line) of the quantile 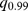 of 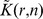 is tested against Monte-Carlo simulations (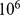 simulations, solid black line) in a 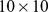 square 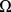 for an increasing number of points 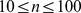. 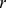 is set at 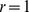. The quantile 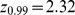 of the standard normal law 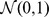 is also represented (black dotted line). C)-Right: Relative errors of CF expansion (grey line) and 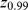 (black dotted line) to Monte-Carlo simulations. The 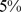 level is represented with a black dotted line. D) Idem to C) for the first percentile 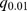 of 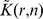 instead of the last one 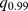.

In Figure 2 A–B, we compare quantiles obtained numerically with Monte-Carlo simulations with the CF expansion (Eq. 5) and the quantiles 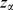 of the standard normal law 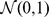 (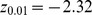 and 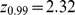). Interestingly, we observe that the CF expansion of 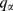 (Eq. 5) with the asymptotical variance (Eq. 3), skewness and kurtosis (Eq. 6–7) is very close to Monte-Carlo simulations with a relative error below 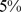 even for 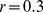, while the normal approximation is not satisfactory with a relative error that is around 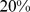 for any 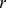, and that reaches 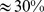 for 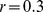 and 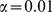. The convergence of the CF expansion is linked to the mean number of pairs of points that are closer than 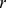, which is 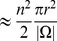. Thus, the number of points 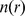 that is needed for the relative error between the CF expansion and Monte-Carlo simulations to be below 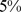 should be approximately given by 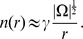 In particular, we found that for 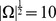 and 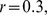 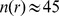 indicating that 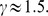


We next investigated the accuracy of CF development for an increasing number of points and a fixed 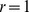. Results are given in Figure 2 C–D. We found that the relative error of the CF development to Monte-Carlo simulations is bounded by 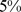 for 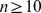 when 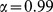 and 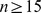 when 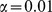, and fall below 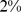 for 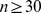 in both cases. Conversely, the relative error to normal approximation reaches 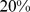 and 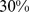 respectively for 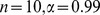 and 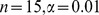, and is above 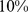 even for 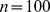. We thus conclude that CF expansion of 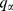 is sufficiently accurate to be used in a large range of 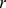 and 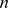 values while the normal approximation does not hold even for intermediate values of 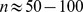. In addition, we highlight that for 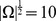 and 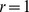, we found that 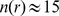, in agreement with 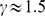.

### Characterizing Objects’ Dispersion and Clusters from 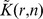 Statistic

To link the statistical deviations of 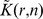 from CSR (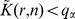 and 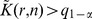, see Figure 1) to quantitative properties of point features, we show here how key features such as the minimal distance between dispersed points or the mean cluster size are related to 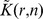 extrema by using standard models of dispersed and clustered processes. While relating the minimum of 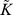 to the distance separating dispersed objects has not been treated, the relation between the maximum of Ripley’s function to the clusters’ size has been recently tackled numerically [12]. In their study, Kiskowski et al. modeled clusters with disk-shape domains with radius 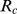 that are regularly separated by a distance 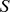, and they used Monte-Carlo simulations where a part 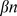 of points was randomly distributed in clusters and 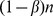 points were distributed outside the clusters. They found that the radius of maximal aggregation 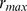 where the Besag L-function 
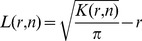
 reaches its maximum was between 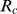 and 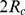 depending on 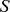.

To extract the minimal distance between objects in a regular pattern from the minimum of 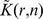 at small distance scale 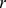, we model the local objects’ organization with a simple inhibition process (chapter 5 [10]), which is a thinned Poisson process (intensity 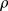) where all pairs of points a distance less than arbitrary 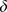 apart would be deleted. Then, the related parametric Ripley’s K function reads ([10], page 72)
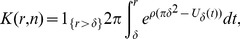
(10)where 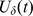 denotes the area of the union of two discs each of radius 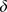 and with centers a distance 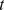 apart, that is [26]: 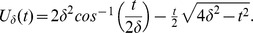 Reinjecting the parametric expression (Eq. 10) of the dispersed 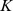 function in 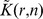 (Eq. 4), we compute the partial derivative 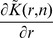 of 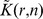 with respect to 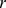 and obtain that 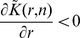 for 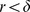 and 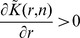 for 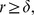 which demonstrates that in an idealized inhibition process, the minimal distance 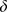 that separates points from each other is equal to 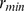 where 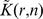 reaches its minimum:



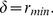
(11)To relate the radius 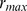 where 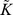 reaches its maximum to the mean clusters’ radius 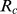, we assume here that clusters’ centers are randomly distributed in 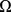 (density 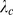), and in that case, the analytical expression of the Ripley’s K function is then given by [27], page 376:
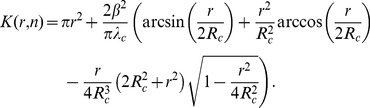
(12)


Reinjecting this parametric expression (Eq.12) in 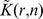, we link the radius 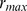 of maximal aggregation with clusters’ radius 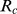 by solving numerically 
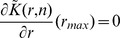
, and find that
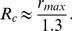
(13)


### Quantitative Analysis of Endocytosis Spatial Organization

**Figure 3:**
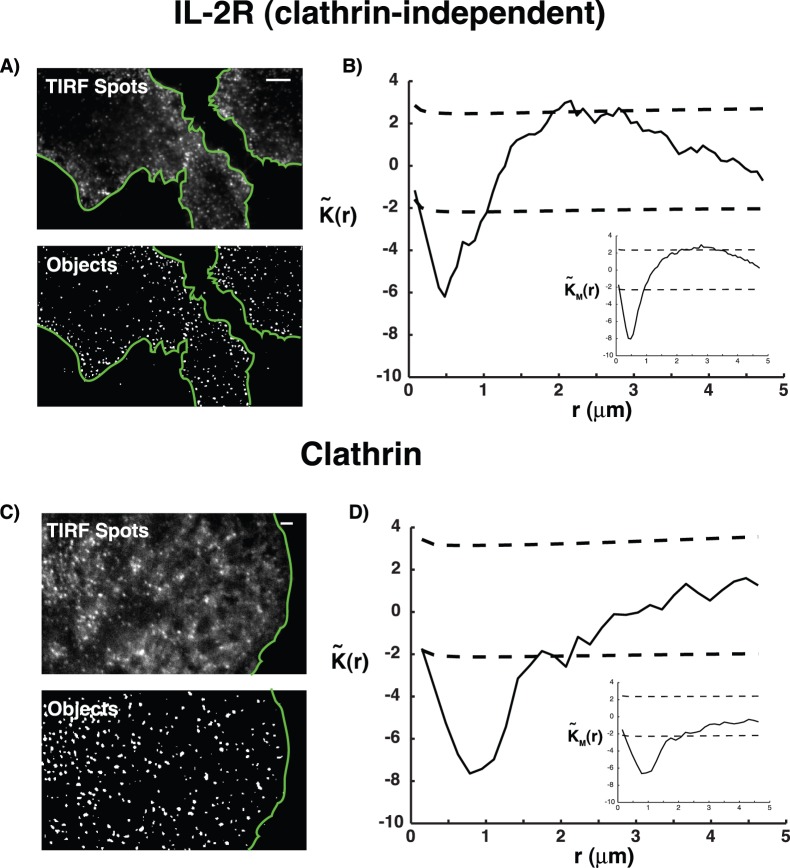
Analysis of endocytosis spatial organization. (A) Clathrin-independent IL-2R putative endocytic sites. Top: IL-2R is labeled with fluorescent antibodies and imaged using total internal reflexion fluorescence (TIRF) microscopy. Bottom: We delimited individual cells by drawing polygonal (green) Regions of Interest (ROIs) in the software Icy [13] (http://icy.bioimageanalysis.org). Positions of putative endocytic sites (objects) inside each cell are then extracted with a multi-scale wavelet analysis [28]. (B) The spatial organization of IL-2R putative endocytic spots is quantified with 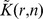 (solid black line). CF expansion of 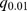 and 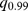 is represented with black dotted lines. In the bottom-right corner, the mean statistic 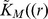 (Eq. 8) is plotted against 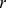 (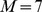 cells, 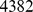 objects)). (C) Clathrin putative endocytic sites. Top: Clathrin light chain is fused with green fluorescent protein (GFP) and imaged using TIRF microscopy. Bottom: Positions of putative endocytic sites are extracted with a multi-scale wavelet analysis [28]. (D) The spatial organization of clathrin putative endocytic spots is quantified with 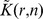 (solid black line). CF expansion of 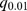 and 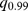 is represented with black dotted lines. In the bottom-right corner, the mean statistic 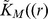 (Eq. 8) is plotted against 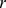 (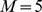 cells, 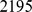 objects). Scale bar = 5 microns.

We analyzed two image data sets representative each of clathrin-dependent (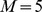 cells, 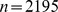 points) and clathrin-independent (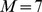 cells, 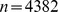 points) pathways. In each case, after extracting the positions of putative endocytic events thanks to a wavelet-based detection [28] (Figure 3 A–C), we computed the modified Ripley’s K function 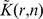 (Eq. (4)) and CF expansions (Eq. (5)) of quantiles 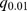 and 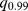 in Figure 3 B–D. We first checked that the characteristic features of 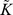 functions were similar whether they were computed on one cell or averaged on several cells (see inserted box in lower corners Figure 3 B–D).

We found that for both pathways, 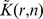 is far below the first percentile 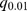 for small 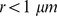 (IL-2R) and 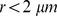 (clathrin), indicating that putative endocytic spots are distributed according to a regular pattern, characterized by a minimum distance between points, which corresponds to the value that minimizes 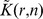. In the case of clathrin-independent pathway, 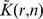 reaches its minimum at 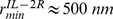 while for clathrin-dependent entry, 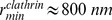. This demonstrates that endocytic sites are non-overlapping and restricted to defined micro-domains with respective radii 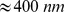 for clathrin-dependent endocytosis and 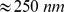 for clathrin-independent pathway.

At higher distance scale 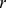, we found that for clathrin-dependent endocytosis, 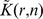 is comprised between the quantiles 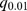 and 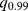 indicating that clathrin spots are homogeneously distributed on the membrane. Repeating our statistical analysis using labeled transferrin, which is the archetypical cargo for internalization through clathrin-mediated endocytosis [29], we got identical profiles to those obtained with clathrin (Figure S1). By contrast, for clathrin-independent pathway, 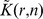 is above 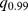 for 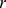 between 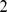 and 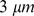 indicating that clathrin-independent spots are partially organized in clusters. Considering that 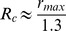 (Eq.13), we deduced that clathrin-independent spots are partially segregated in clusters with radius 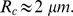


## Discussion

We have developed a new test statistic based on the Ripley’s K function that facilitates the quantitative analysis of the spatial organization of point patterns at multiple scales. This test allows us to statistically assess the presence of specific point patterns such as point clusters or dispersion with no need for resampling by providing an asymptotic closed-form expression of the critical quantiles of the Ripley’s K function under spatial randomness. In addition, we related the extrema of our statistics to the geometrical properties of the observed patterns by using standard models of dispersed and clustered point patterns.

We applied our method to study the spatial organization of molecules implicated in different endocytosis pathways, and we found that the spatial organization of endocytosis was different upon the mechanism (dependent or independent of clathrin), which might reflect distinct cellular functions of each pathway. We note that all clathrin and IL-2R spots are not necessarily entering the cell, as some spots might disassemble or detach before being endocytosed [30]. It would thus be interesting to couple our statistical analysis with live cell imaging to compare the spatial organization of real endocytic events and abortive ones.

A major difficulty in Ripley-based statistical tests is their interpretation when the null hypothesis of objects’ random distribution is rejected. In particular for IL-2R receptors, the detected aggregation could result either from small clusters, or from a very local increase of the receptors’ density near the cell boundary, or from a mixture of both. We have thus repeated our analysis by eroding the cell’s contour mask by 300 nm (isotropic ball of radius 3 pixels) and 500 nm (5 pixels) to test boundary effects. Interestingly, we found profiles very similar to those obtained with the whole cell (Figure S2) with a maximum of the Ripley’s K function reached for 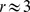 microns as above. We thus conclude that the local increase of IL-2R receptors at cell boundary does not have much impact on the behavior of Ripley’s K function and that receptors are truly organized in clusters with a radius of 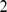 microns.

In this study, we developed a robust and fast analytical method to test whether an objects’ distribution deviates from CSR. A promising extension would be to test whether the spatial organization of points can be described with some specific parametric models, in particular the large classes of Neyman-Scott [27], [31] or Strauss [27], [32] processes. This would open the door to analytical comparison of points’ distributions against each other through embedding and statistical learning.

## Materials and Methods

### Experimental Protocol, TIRF Microscopy

For clathrin-independent endocytosis, Hep2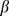 cells (1×105) expressing IL-2R were incubated 2 min with anti-IL-2R coupled to Cy3 fluorochrome in a TIRF medium (25 mM Hepes, 135 mM NaCl, 5 mM KCl, 1.8 mM CaCl2, 0.4 mM MgCl2, 4.5 g/L glucose, pH 7.4 and 0.5% BSA) at 37 C and washed. For clathrin-dependent endocytosis BSC-1 cells, expressing clathrin-light chain fused to GFP were used. Cells were incubated in an environmental control system set to 37 C and movies of 100 s at 1Hz were acquired. Experiments were performed using a TIRF microscope (IX81F-3, Olympus) equipped with a 100x NA 1.45 Plan Apo TIRFM Objective (Olympus) and fully controlled by CellM (Olympus).

### Quantitative Image Analysis

We first delimited cells’ contours by drawing polygonal Region of Interest (ROIs) with the Icy software [13] (http://icy.bioimageanalysis.org). We then used a wavelet-based detection method [28], implemented as a plugin *Spot detector* in Icy to extract the two dimensional positions of putative endocytic spots at the cellular membrane. In the clathrin-independent pathway, a part of IL-2R spots diffused at the cell membrane and we extracted the signal corresponding to static spots entering the cell by first stacking time sequences in a single image (mean), and by then applying our wavelet-based detection algorithm on the stacked image.

## Supporting Information

Figure S1
**Analysis of the spatial organization of transferrin endocytosis.** (A) Clathrin-dependent transferrin putative endocytic sites. Top: Transferrin is labeled with fluorescent antibodies and imaged using total internal reflexion fluorescence (TIRF) microscopy. Bottom: We delimited manually individual cells by drawing polygonal (green) Regions of Interest (ROIs) in the software Icy [13] (http://icy.bioimageanalysis.org). Positions of putative endocytic sites (objects) inside each cell are then extracted with a multi-scale wavelet analysis [28]. (B) The spatial organization of Transferrin putative endocytic spots is quantified with the mean statistic 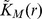 (Eq. (8) of the main manuscript, 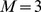 cells (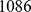 objects), solid black line). Cornish-Fisher expansion of 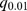 and 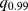 (Eq. (5) of the main manuscript) are represented with black dotted lines.(EPS)

Figure S2
**Analysis of the spatial organization of clathrin-independent endocytosis with erosions of the cell’s contour mask.** We have tested the impact of the local accumulation of IL-2R spots at the cell boundary by eroding the cell’s contour mask by 300 nm (isotropic ball of radius 3 pixels) and 500 nm (5 pixels) to test boundary effects. The spatial organization of IL-2R putative endocytic spots is quantified with the mean statistic 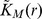 (Eq. (8) of the main manuscript, 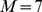 cells (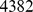 objects)) for no erosion (black line), 300 nm-erosion (blue line) and 500 nm-erosion (green line). Cornish-Fisher expansion of 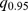 and 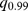 (Eq. (5) of the main manuscript) are represented with black dotted lines.(EPS)

File S1 **Supplementary Methods** Detailed computations of the skewness and the kurtosis of the Ripley’s K function.
(PDF)
