# Supplementary material for: Analysis of the Spatial Organization of Molecules with Robust Statistics
Source: PLoS One. 2013 Dec 4;8(12):e80914. doi: 10.1371/journal.pone.0080914 (PMC3857798; doi:10.1371/journal.pone.0080914)
Supplement: File S1 — Supplementary Methods Detailed computations of the skewness and the kurtosis of the Ripley’s K function. (PDF) [file pone.0080914.s003.pdf]

Analysis of the spatial organization of molecules with robust statistics: Supplementary Material

Thibault Lagache[1,2,*], Gabriel Lang[3], Nathalie Sauvonnet[4,2], Jean-Christophe Olivo-Marin [1,2,*]
1 Unité d'Analyse d'Images Quantitative, Institut Pasteur. Paris, France
2 Unité de Recherche Associée 2582, Centre National de la Recherche Scientifique. Paris, France
3 Unité Mixte de Recherche 518 Mathématiques et Informatique Appliquées, AgroParisTech and INRA. Paris, France
4 Unité de Biologie des Interactions Cellulaires, Institut Pasteur. Paris, France
$*$ E-mail: thibault.lagache@pasteur.fr, jcolivo@pasteur.fr

In this supplementary material, our goal is to compute the four first moment of the Ripley's K-function $K(r,n)$. Computation of $\mathbb{E}\{K(r,n)\}$ and $\mathrm{var}\{K(r,n)\}$ can be found in [1], however we refine here the computation of $\mathrm{var}\{K(r,n)\}$ for a small number of points in section 2 and we reproduce the computation of $\mathbb{E}\{K(r,n)\}$ for sake of clarity in section 1. Finally, the third and the fourth moments of $K(r,n)$, $\mathbb{E}\left\{(K(r,n)-\mathbb{E}\{K(r,n)\})^3\right\}$ and $\mathbb{E}\left\{(K(r,n)-\mathbb{E}\{K(r,n)\})^4\right\}$, are computed in sections 3 and 4.

1 computation of $\mathbb{E}\{K(r,n)\}$

Denoting $\psi(\mathbf{x},\mathbf{y}) = \mathbf{1}_{\{|\mathbf{x}-\mathbf{y}|\leq r\}}k(\mathbf{x},\mathbf{y})$, we decompose the symmetric function $\phi(\mathbf{x},\mathbf{y})$ as

$$\phi(\mathbf{x},\mathbf{y}) = \frac{1}{2}\left(\psi(\mathbf{x},\mathbf{y}) + \psi(\mathbf{y},\mathbf{x})\right), \tag{1}$$

and we re-write $K(r,n)$ as

$$K(r,n) = \frac{a}{n(n-1)}\sum_{\mathbf{x}\neq\mathbf{y}}\phi(\mathbf{x},\mathbf{y}) = \frac{a}{n(n-1)}\sum_{\mathbf{x}\neq\mathbf{y}}\frac{1}{2}\left(\psi(\mathbf{x},\mathbf{y}) + \psi(\mathbf{y},\mathbf{x})\right). \tag{2}$$

Then, assuming a uniform distribution of points in Ω and denoting

$$\alpha_r = n(n-1)\dots(n-r+1)a^{-r}\mu_{2r}, \tag{3}$$

where μ_{2r} is the Lebesgue measure on $\mathbb{R}^{2r}$, we have [1]

$$\mathbb{E}\{K(r,n)\} = \frac{a}{n(n-1)}\int_{\Omega^2}\phi(\mathbf{x},\mathbf{y})d\alpha_2(\mathbf{x},\mathbf{y}). \tag{4}$$

that is

$$\mathbb{E}\{K(r,n)\} = \frac{a}{n(n-1)}\int_{\Omega^2}\psi(\mathbf{x},\mathbf{y})d\alpha_2(\mathbf{x},\mathbf{y}). \tag{5}$$

Denoting

$$I_0 = \int_{\Omega^2}\psi(\mathbf{x},\mathbf{y})d(\mathbf{x},\mathbf{y}) = \int_{\Omega^2}\mathbf{1}_{\{|\mathbf{x}-\mathbf{y}|\leq r\}}k(\mathbf{x},\mathbf{y})d(\mathbf{x},\mathbf{y}), \tag{6}$$

we re-write

$$\mathbb{E}\{K(r,n)\} = \frac{I_0}{a}, \tag{7}$$

and we further compute I_0 by considering local polar coordinates $\mathbf{y}(r_\mathbf{y},\theta_\mathbf{y})$ around $\mathbf{x}(0,0)$ in Ω, with $0\leq r_\mathbf{y}\leq r$ and $-\dfrac{\Theta(r_\mathbf{y})}{2}\leq\theta_\mathbf{y}\leq\dfrac{\Theta(r_\mathbf{y})}{2}$ where $\Theta(r_\mathbf{y})$ is the part of the perimeter $b(\mathbf{x},r_\mathbf{y})$ that is in Ω:

$$\left(2\pi-\Theta(r_\mathbf{y})\right)r_\mathbf{y} = |\partial b(\mathbf{x},r_\mathbf{y})\cap\Omega|, \tag{8}$$

that is

$$2\pi-\Theta(r_\mathbf{y}) = \frac{|\partial b(\mathbf{x},r_\mathbf{y})\cap\Omega|}{r_\mathbf{y}} = \frac{2\pi}{k(\mathbf{x},\mathbf{y})}. \tag{9}$$

We then have

$$I_0 = \int_\Omega \int_0^r 2\pi r_{\mathbf{y}} dr_{\mathbf{y}} d\mathbf{x} = a\pi r^2. \tag{10}$$

Finally, reinjecting I_0 (Eq. (10)) in Eq. (7) we have

$$\mathbb{E}\{K(r,n)\} = \frac{I_0}{a} = \pi r^2. \tag{11}$$

Integrals $(I_j)_{j\geq 0}$ that are introduced all along this supplementary material are summarized with their numerical value in Supplementary Table **S1**.

2 computation of var $\{K(r,n)\} = \mathbb{E}\left\{(K(r,n) - \mathbb{E}\{K(r,n)\})^2\right\}$

We first introduce the centered ($\mathbb{E} = 0$) estimator $K_0(r,n)$:

$$K_0(r,n) = K(r,n) - \mathbb{E}\{K(r,n)\} = \frac{a}{n(n-1)} \sum_{\mathbf{x}\neq\mathbf{y}} \phi_0(\mathbf{x},\mathbf{y}), \tag{12}$$

where

$$\phi_0(\mathbf{x},\mathbf{y}) = \phi(\mathbf{x},\mathbf{y}) - \frac{\pi r^2}{a}. \tag{13}$$

We then have

$$\text{var}\{K(r,n)\} = \mathbb{E}\left\{K_0^2(r,n)\right\} = \frac{a^2}{(n(n-1))^2}\mathbb{E}\left\{\left(\sum_{\mathbf{x}\neq\mathbf{y}} \phi_0(\mathbf{x},\mathbf{y})\right)^2\right\}, \tag{14}$$

that we expand as

$$\left(\sum_{\mathbf{x}\neq\mathbf{y}} \phi_0(\mathbf{x},\mathbf{y})\right)^2 = a_2 \sum_{\mathbf{x}\neq\mathbf{y}} \phi_0^2(\mathbf{x},\mathbf{y}) + a_3 \sum_{\mathbf{x}\neq\mathbf{y}\neq\mathbf{z}} \phi_0(\mathbf{x},\mathbf{y})\phi_0(\mathbf{x},\mathbf{z}) + a_4 \sum_{\mathbf{x}\neq\mathbf{y}\neq\mathbf{z}\neq\mathbf{w}} \phi_0(\mathbf{x},\mathbf{y})\phi_0(\mathbf{z},\mathbf{w}). \tag{15}$$

Computing the number of terms in each sum of the equation above, we obtain that

$$(n(n-1))^2 = a_2 n(n-1) + a_3 n(n-1)(n-2) + a_4 n(n-1)(n-2)(n-3), \tag{16}$$

that is

$$n^4 - 2n^3 + n^2 = a_4 n^4 + (a_3 - 6a_4)n^3 + (a_2 - 3a_3 + 11a_4)n^2 + (-a_2 + 2a_3 - 6a_4). \tag{17}$$

Identifying polynomial coefficients, we obtain that

$$a_2 = 2, a_3 = 4, \text{ and } a_4 = 1, \tag{18}$$

leading to

$$\text{var}\{K(r,n)\} = \frac{a^2}{(n(n-1))^2}\left(2\mathbb{E}\left\{\sum_{\mathbf{x}\neq\mathbf{y}} \phi_0^2(\mathbf{x},\mathbf{y})\right\} + 4\mathbb{E}\left\{\sum_{\mathbf{x}\neq\mathbf{y}\neq\mathbf{z}} \phi_0(\mathbf{x},\mathbf{y})\phi_0(\mathbf{x},\mathbf{y})\right\} + \mathbb{E}\left\{\sum_{\mathbf{x}\neq\mathbf{y}\neq\mathbf{z}\neq\mathbf{w}} \phi_0(\mathbf{x},\mathbf{y})\phi_0(\mathbf{z},\mathbf{w})\right\}\right). \tag{19}$$

Considering that $\mathbf{x}$ and $\mathbf{y}$ are uniformly distributed in Ω, Eq. (19) becomes

$$\begin{aligned} \mathrm{var}\{K(r,n)\} &= \frac{a^2}{(n(n-1))^2}\Big(2\int_{\Omega^2}\phi_0^2(\mathbf{x},\mathbf{y})d\alpha_2(\mathbf{x},\mathbf{y}) \\ &+ 4\int_{\Omega^3}\phi_0(\mathbf{x},\mathbf{y})\phi_0(\mathbf{x},\mathbf{w})d\alpha_3(\mathbf{x},\mathbf{y},\mathbf{w}) + \int_{\Omega^4}\phi_0(\mathbf{x},\mathbf{y})\phi_0(\mathbf{w},\mathbf{z})d\alpha_4(\mathbf{x},\mathbf{y},\mathbf{w},\mathbf{z})\Big) \end{aligned} \tag{20}$$

where α_r is given by Eq. (3). Because

$$\int_{\Omega^2}\phi_0(\mathbf{x},\mathbf{y})d\alpha_2(\mathbf{x},\mathbf{y}) = \int_{\Omega^2}\phi(\mathbf{x},\mathbf{y})d\alpha_2(\mathbf{x},\mathbf{y}) - a\pi r^2 = 0, \tag{21}$$

we have

$$\int_{\Omega^4}\phi_0(\mathbf{x},\mathbf{y})\phi_0(\mathbf{w},\mathbf{z})d\alpha_4(\mathbf{x},\mathbf{y},\mathbf{w},\mathbf{z}) = \alpha_4\int_{\Omega^2}\phi_0(\mathbf{x},\mathbf{y})d(\mathbf{x},\mathbf{y})\int_{\Omega^2}\phi_0(\mathbf{w},\mathbf{z})d(\mathbf{w},\mathbf{z}) = 0. \tag{22}$$

Then, denoting $\beta = \frac{\pi r^2}{a}$, we expand the two remaining integrals of Eq. (46)

$$\phi_0(\mathbf{x},\mathbf{y})^2 = \phi(\mathbf{x},\mathbf{y})^2 - 2\beta\phi(\mathbf{x},\mathbf{y}) + \beta^2, \tag{23}$$

and

$$\phi_0(\mathbf{x},\mathbf{y})\phi_0(\mathbf{x},\mathbf{z}) = \phi(\mathbf{x},\mathbf{y})\phi(\mathbf{x},\mathbf{z}) - \beta\left(\phi(\mathbf{x},\mathbf{y}) + \phi(\mathbf{x},\mathbf{z})\right) + \beta^2, \tag{24}$$

Because $\int_{\Omega^2}\phi(\mathbf{x},\mathbf{y}) = a\pi r^2 = a^2\beta$, we have

$$\int_{\Omega^2}\phi_0^2(\mathbf{x},\mathbf{y})d(\mathbf{x},\mathbf{y}) = \int_{\Omega^2}\phi(\mathbf{x},\mathbf{y})^2 - 2\beta\phi(\mathbf{x},\mathbf{y}) + \beta^2 d(\mathbf{x},\mathbf{y}) = \int_{\Omega^2}\phi(\mathbf{x},\mathbf{y})^2 - a^2\beta^2, \tag{25}$$

and

$$\int_{\Omega^3}\phi_0(\mathbf{x},\mathbf{y})\phi_0(\mathbf{x},\mathbf{z})d(\mathbf{x},\mathbf{y},\mathbf{z}) = \int_{\Omega^2}\phi(\mathbf{x},\mathbf{y})\phi(\mathbf{x},\mathbf{z})d(\mathbf{x},\mathbf{y},\mathbf{z}) - a^3\beta^2. \tag{26}$$

Consequently, we are now left with the computations of the integrals

$$I_1 = \int_{\Omega^2}\phi(\mathbf{x},\mathbf{y})^2 d(\mathbf{x},\mathbf{y}) \tag{27}$$

and

$$I_2 = \int_{\Omega^3}\phi(\mathbf{x},\mathbf{y})\phi(\mathbf{x},\mathbf{z})d(\mathbf{x},\mathbf{y},\mathbf{z}). \tag{28}$$

We first observe that for points $\mathbf{x}$ that are at a distance $|\mathbf{x}-\partial\Omega| > 2r$ from the domain boundary, there is no edge correction for any points $\mathbf{y},\mathbf{z}$ inside the domain Ω: $k(\mathbf{x},\mathbf{y}) = k(\mathbf{y},\mathbf{x}) = \mathbf{1}_{\{|\mathbf{x}-\mathbf{y}|<r\}}$. Consequently, integrals I_1 and I_2 can be decomposed as follows

$$\begin{aligned} I_1 &= \int_{\Omega^2}\mathbf{1}_{\{|\mathbf{x}-\partial\Omega|>2r\}}\mathbf{1}_{\{|\mathbf{x}-\mathbf{y}|<r\}}d(\mathbf{x},\mathbf{y}) \\ &+ \frac{1}{4}\int_{\Omega^2}\mathbf{1}_{\{|\mathbf{x}-\partial\Omega|<2r\}}\mathbf{1}_{\{|\mathbf{x}-\mathbf{y}|<r\}}\left(k(\mathbf{x},\mathbf{y}) + k(\mathbf{y},\mathbf{x})\right)^2 d(\mathbf{x},\mathbf{y}) \end{aligned} \tag{29}$$

and,

$$\begin{aligned} I_2 &= \int_{\Omega^3}\mathbf{1}_{\{|\mathbf{x}-\partial\Omega|>2r\}}\mathbf{1}_{\{|\mathbf{x}-\mathbf{y}|<r\}}\mathbf{1}_{\{|\mathbf{x}-\mathbf{z}|<r\}}d(\mathbf{x},\mathbf{y},\mathbf{z}) \\ &+ \frac{1}{4}\int_{\Omega^3}\mathbf{1}_{\{|\mathbf{x}-\partial\Omega|<2r\}}\mathbf{1}_{\{|\mathbf{x}-\mathbf{y}|<r\}}\mathbf{1}_{\{|\mathbf{x}-\mathbf{z}|<r\}}\left(k(\mathbf{x},\mathbf{y}) + k(\mathbf{y},\mathbf{x})\right)\left(k(\mathbf{x},\mathbf{z}) + k(\mathbf{z},\mathbf{x})\right)d(\mathbf{x},\mathbf{y},\mathbf{z}). \end{aligned} \tag{30}$$

Because $\int_\Omega \mathbf{1}_{\{\mathbf{x}-\partial\Omega|>2r\}} d\mathbf{x} = |\Omega| - 2r|\partial\Omega| = a - 2ur$, where $u = |\partial\Omega|$ is the perimeter of the domain Ω, and that for all $\mathbf{x}$, such that $|\mathbf{x} - \partial\Omega| > 2r$, $\int_\Omega \mathbf{1}_{\{|\mathbf{x}-\mathbf{y}|<r\}} d\mathbf{y} = \pi r^2$, we have

$$\int_{\Omega^2} \mathbf{1}_{\{|\mathbf{x}-\partial\Omega|>2r\}} \mathbf{1}_{\{|\mathbf{x}-\mathbf{y}|<r\}} d(\mathbf{x},\mathbf{y}) = (a - 2ur)\pi r^2, \tag{31}$$

and

$$\int_{\Omega^3} \mathbf{1}_{\{|\mathbf{x}-\partial\Omega|>2r\}} \mathbf{1}_{\{|\mathbf{x}-\mathbf{y}|<r\}} \mathbf{1}_{\{|\mathbf{x}-\mathbf{y}|<r\}} d(\mathbf{x},\mathbf{y},\mathbf{z}) = (a - 2ur) \left(\pi r^2\right)^2. \tag{32}$$

Consequently, denoting $A_h = \{\mathbf{y} \in \Omega \text{ such that } |\mathbf{x} - \mathbf{y}| < r \text{ given that } |\mathbf{x} - \partial\Omega| = h\}$, I_1 and I_2 reduce to

$$I_1 = (a - 2ur)\pi r^2 + \frac{u}{4}\int_0^{2r}\int_{A_h} \left(k(h,\mathbf{y}) + k(\mathbf{y},h)\right)^2 d\mathbf{y}dh \tag{33}$$

and,

$$I_2 \quad = \quad (a - 2ur)\left(\pi r^2\right)^2 + \frac{u}{4}\int_0^{2r}\left(\int_{A_h} \left(k(h,\mathbf{y}) + k(\mathbf{y},h)\right) d\mathbf{y}\right)^2 dh. \tag{34}$$

Assuming that the edge of the domain boundary $\partial\Omega$ is straight where it intersects $b(\mathbf{x}, |\mathbf{x} - \mathbf{y}|)$, $k(h,\mathbf{y})$ and $k(\mathbf{y},h)$ can be determined analytically [2], and are given by:

$$\begin{aligned} k(h,\mathbf{y}) &\approx \left(1 - \frac{1}{\pi}\arccos\left(\frac{\min(|\mathbf{x}-\mathbf{y}|, h)}{|\mathbf{x}-\mathbf{y}|}\right)\right)^{-1}, \text{ and,} \\ k(\mathbf{y},h) &\approx \left(1 - \frac{1}{\pi}\arccos\left(\frac{\min(|\mathbf{x}-\mathbf{y}|, |\mathbf{y}-\partial\Omega|)}{|\mathbf{x}-\mathbf{y}|}\right)\right)^{-1}. \end{aligned} \tag{35}$$

However, using analytical expressions (35) in Eq. (33)-(34) does not lead to closed form expressions for I_1 and I_2. We thus use a finite difference algorithm with respect to the variable h ($n_h = \frac{2r}{dh}$ steps of size $dh = 0.001$) coupled with a Monte-Carlo sampling of $\mathbf{y}$ in each $A_{h_j=j.dh}$, $1 \le j \le n_h$ ($n_\mathbf{y} = 1000$ random draws $\mathbf{y}_i$, $1 \le i \le n$), and approximate

$$\int_0^{2r}\int_{A_h} \left(k(h,\mathbf{y}) + k(\mathbf{y},h)\right)^2 d\mathbf{y}dh \approx \sum_{j=1}^{n_h} |A_{h_j}| \frac{1}{n_\mathbf{y}} \sum_{i}^{n_\mathbf{y}} \left(k(h_j,\mathbf{y}_i) + k(\mathbf{y}_i,h_j)\right)^2 dh \tag{36}$$

and

$$\int_0^{2r}\left(\int_{A_h} \left(k(h,\mathbf{y}) + k(\mathbf{y},h)\right) d\mathbf{y}\right)^2 dh \approx \sum_{j=1}^{n_h}\left(|A_{h_j}| \frac{1}{n_\mathbf{y}} \sum_{i}^{n_\mathbf{y}} \left(k(h_j,\mathbf{y}_i) + k(\mathbf{y}_i,h_j)\right)\right)^2 dh \tag{37}$$

with [3]

$$|A_{h_j}| = \pi r^2 \left(1 - \frac{1}{\pi}\left(\arccos\left(\frac{h_j}{r}\right) + \frac{h_j}{r}\sqrt{1 - \left(\frac{h_j}{r}\right)^2}\right)\right). \tag{38}$$

Finally, we obtain following numerical approximations

$$\frac{u}{2}\int_0^{2r}\int_{A_h} \left(k(h,\mathbf{y}) + k(\mathbf{y},h)\right)^2 d\mathbf{y}dh \approx ru(\pi r^2)2.305, \tag{39}$$

and

$$\frac{u}{4}\int_0^{2r}\left(\int_{A_h} \left(k(h,\mathbf{y}) + k(\mathbf{y},h)\right) d\mathbf{y}\right)^2 dh \approx ru(\pi r^2)^2 2.0066. \tag{40}$$

Reinjecting approximations (39) and (40) in (33) and (34), we have

$$I_1 = a^2\beta\left(1+0.305\frac{ur}{a}\right) \text{ and } I_2 = a^3\beta^2\left(1+0.0066\frac{ur}{a}\right), \tag{41}$$

leading to

$$\int_{\Omega^2}\phi_0^2(\mathbf{x},\mathbf{y})d(\mathbf{x},\mathbf{y}) = I_1 - a^2\beta^2 = a^2\left(\beta\left(1+0.305\frac{ur}{a}\right)-\beta^2\right), \tag{42}$$

and

$$\int_{\Omega^3}\phi_0(\mathbf{x},\mathbf{y})\phi_0(\mathbf{x},\mathbf{z})d(\mathbf{x},\mathbf{y},\mathbf{z}) = I_2 - a^3\beta^2 = a^3\beta^2\left(0.0066\frac{ur}{a}\right). \tag{43}$$

Finally, we obtain

$$\begin{aligned}\operatorname{var}\{K(r,n)\} &= \frac{a^2}{(n(n-1))^2}\left\{2\int_{\Omega^2}\phi_0^2(\mathbf{x},\mathbf{y})d\alpha_2(\mathbf{x},\mathbf{y}) + 4\int_{\Omega^3}\phi_0(\mathbf{x},\mathbf{y})\phi_0(\mathbf{x},\mathbf{w})d\alpha_3(\mathbf{x},\mathbf{y},\mathbf{w})\right\} \\ &= \frac{2a^2}{n(n-1)}\left(\beta\left(1+0.305\frac{ur}{a}\right)-\beta^2+2(n-2)\beta^2\left(0.0066\frac{ur}{a}\right)\right) \end{aligned} \tag{44}$$

that is

$$\operatorname{var}\{K(r,n)\} = \frac{2a^2}{n(n-1)}\left(\beta\left(1+0.305\frac{ur}{a}\right)+\beta^2\left(-1+2(n-2)0.0066\frac{ur}{a}\right)\right). \tag{45}$$

which reduces for $n \gg 1$ to

$$\operatorname{var}\{K(r,n)\} \approx \frac{2}{\lambda^2}\left(\beta\left(1+0.305\frac{ur}{a}\right)+\beta^2\left(-1+0.0132\right)\frac{ur}{a}\right). \tag{46}$$

where $\lambda = \frac{n}{a}$ is the empirical density of points. Formula (46) is in agreement with [1], page 40.

3 computation of $\mathbb{E}\left\{(K(r,n)-\mathbb{E}\{K(r,n)\})^3\right\}$

Using $\phi_0(\mathbf{x},\mathbf{y}) = \phi(\mathbf{x},\mathbf{y}) - \beta$, we have

$$\mathbb{E}\left\{(K(r,n)-\mathbb{E}\{K(r,n)\})^3\right\} = \frac{a^3}{(n(n-1))^3}\mathbb{E}\left\{\left(\sum_{\mathbf{x}\neq\mathbf{y}}\phi_0(\mathbf{x},\mathbf{y})\right)^3\right\}. \tag{47}$$

We expand $\left(\sum_{\mathbf{x}\neq\mathbf{y}}\phi_0(\mathbf{x},\mathbf{y})\right)^3$ as

$$\left(\sum_{\mathbf{x}\neq\mathbf{y}}\phi_0(\mathbf{x},\mathbf{y})\right)^3 = \sum_{j=2}^{6} S_j \tag{48}$$

where S_j is the sum of the terms containing j different points:

$$\begin{aligned} S_2 &= a_2\sum_{\mathbf{x}\neq\mathbf{y}}\phi_0^3(\mathbf{x},\mathbf{y}) \\ S_3 &= a_3^1\sum_{\mathbf{x}\neq\mathbf{y}\neq\mathbf{z}}\phi_0^2(\mathbf{x},\mathbf{y})\phi_0(\mathbf{x},\mathbf{z}) + a_3^2\sum_{\mathbf{x}\neq\mathbf{y}\neq\mathbf{z}}\phi_0(\mathbf{x},\mathbf{y})\phi_0(\mathbf{x},\mathbf{z})\phi_0(\mathbf{y},\mathbf{z}), \\ S_4 &= a_4^1\sum_{\mathbf{x}\neq\mathbf{y}\neq\mathbf{z}\neq\mathbf{w}}\phi_0^2(\mathbf{x},\mathbf{y})\phi_0(\mathbf{z},\mathbf{w}) + a_4^2\sum_{\mathbf{x}\neq\mathbf{y}\neq\mathbf{z}\neq\mathbf{w}}\phi_0(\mathbf{x},\mathbf{y})\phi_0(\mathbf{x},\mathbf{z})\phi_0(\mathbf{x},\mathbf{w}) \\ &+ a_4^3\sum_{\mathbf{x}\neq\mathbf{y}\neq\mathbf{z}\neq\mathbf{w}}\phi_0(\mathbf{x},\mathbf{y})\phi_0(\mathbf{y},\mathbf{z})\phi_0(\mathbf{z},\mathbf{w}), \\ S_5 &= a_5\sum_{\mathbf{x}\neq\mathbf{y}\neq\mathbf{z}\neq\mathbf{w}\neq\mathbf{r}}\phi_0(\mathbf{x},\mathbf{y})\phi_0(\mathbf{z},\mathbf{w})\phi_0(\mathbf{x},\mathbf{r}), \\ \text{and } S_6 &= a_6\sum_{\mathbf{x}\neq\mathbf{y}\neq\mathbf{z}\neq\mathbf{w}\neq\mathbf{r}\neq\mathbf{u}}\phi_0(\mathbf{x},\mathbf{y})\phi_0(\mathbf{z},\mathbf{w})\phi_0(\mathbf{r},\mathbf{u}), \end{aligned} \tag{49}$$

and we then have

$$\mathbb{E}\left\{(K(r,n)-\mathbb{E}\{K(r,n)\})^3\right\}=\frac{a^3}{(n(n-1))^3}\sum_{j=2}^{6}\mathbb{E}\{S_j\}. \tag{50}$$

We are thus left with the computations of each mean $\mathbb{E}(S_j)$, for $2 \leq j \leq 6$. We begin with the computation of multiplicative coefficients $a_2, a_3^1, a_3^2 \ldots a_5$ and a_6 in following sub-section 3.1, and will perform the computations of each term $\mathbb{E}(S_j)$ in sub-section 3.2.

3.1 computation of coefficients $a_2, a_3^1, \ldots a_6$

Computing the number of terms appearing in each sum of Eq. (48), we obtain

$$(n(n-1))^3 = a_2\tilde{\alpha}_2 + \sum_{j=1}^{2} a_3^j\tilde{\alpha}_3 + \sum_{j=1}^{3} a_4^j\tilde{\alpha}_4 + a_5\tilde{\alpha}_5 + a_6\tilde{\alpha}_6, \tag{51}$$

where $\tilde{\alpha}_i = n(n-1)\ldots(n-i+1)$ is the number of ways to choose an ordered subset of i points among n. Expanding Eq. (51), we obtain

$$\begin{aligned} n^6-3n^5+3n^4-n^3 &= a_6n^6+(-16a_6+a_5)n^5+(85a_6-10a_5+\sum_{j=1}^{3}a_4^j)n^4 \\ &+ (-225a_6+35a_5-6\sum_{j=1}^{3}a_4^j+\sum_{j=1}^{2}a_3^j)n^3 \\ &+ (274a_6-50a_5+11\sum_{j=1}^{3}a_4^j-3\sum_{j=1}^{2}a_3^j+a_2)n^2 \\ &+ (-120+24a_5-6\sum_{j=1}^{3}a_4^j+2\sum_{j=1}^{2}a_3^j-a_2)n, \end{aligned} \tag{52}$$

Identifying polynomial coefficients in Eq. (52), we have

$$a_2=4, \sum_{j=1}^{2}a_3^j=32, \sum_{j=1}^{3}a_4^j=38, a_5=12 \text{ and } a_6=1. \tag{53}$$

Then, because $\phi_0(\mathbf{x},\mathbf{y})$ is a symmetric function there is $3\times 2^3=24$ ways of writing $\phi_0^2(\mathbf{x},\mathbf{y})\phi_0(\mathbf{x},\mathbf{z})$. Indeed, there is 3 possible positions for $\phi_0(\mathbf{x},\mathbf{z})$ and 2 ways to write each symmetric term. Consequently, $a_3^1=24$, and $a_3^2=\sum_{j=1}^{2}a_3^j-a_3^1=8$. Similarly, there is $3\times 2^3=24$ ways of writing $\phi_0^2(\mathbf{x},\mathbf{y})\phi_0(\mathbf{z},\mathbf{w})$, but in that case, the expression is symmetric in $\mathbf{x}$ and $\mathbf{y}$ as well as in $\mathbf{z}$ and $\mathbf{w}$. Consequently, $\phi_0^2(\mathbf{x},\mathbf{y})\phi_0(\mathbf{z},\mathbf{w})$ is counted 4 times in $\sum_{\mathbf{x}\neq\mathbf{y}}\sum_{\mathbf{z}\neq\mathbf{w}}\phi_0^2(\mathbf{x},\mathbf{y})\phi_0(\mathbf{z},\mathbf{w})$ and $a_4^1=24/4=6$. Concerning a_4^2, there is $6\times 2^3=48$ ways of writing $\phi_0(\mathbf{x},\mathbf{y})\phi_0(\mathbf{x},\mathbf{z})\phi_0(\mathbf{x},\mathbf{w})$ and the expression is symmetric in $\mathbf{y}$, $\mathbf{w}$ and $\mathbf{z}$ leading to $a_4^2=48/6=8$. Finally, there is 48 ways of writing $\phi_0(\mathbf{x},\mathbf{y})\phi_0(\mathbf{y},\mathbf{z})\phi_0(\mathbf{z},\mathbf{w})$ and the symmetric role of the couple of points $(\mathbf{x},\mathbf{y})$ and $(\mathbf{z},\mathbf{w})$ leads to $a_4^3=24$. We can check here that $a_4^1+a_4^2+a_4^3=38$.

3.2 computation of $\mathbb{E}\{S_j\}$ for $2\leq j\leq 6$

3.2.1 computation of $\mathbb{E}\{S_2\}$

First, assuming a uniform distribution of the points inside Ω and using $\alpha_r=n(n-1)\ldots(n-r+1)a^{-r}\mu_{2r}$, we have

$$\mathbb{E}\{S_2\}=4\int_{\Omega^2}\phi_0^3(\mathbf{x},\mathbf{y})d\alpha_2(\mathbf{x},\mathbf{y}), \tag{54}$$

that we expand as

$$\begin{aligned}\mathbb{E}\left\{S_2\right\} &= 4\alpha_2 \int_{\Omega^2} \left(\phi^3(\mathbf{x},\mathbf{y}) - 3\beta\phi^2(\mathbf{x},\mathbf{y}) + 3\beta^2\phi(\mathbf{x},\mathbf{y}) - \beta^3\right) d(\mathbf{x},\mathbf{y}) \\ &= 4\alpha_2 \left(\int_{\Omega^2} \phi^3(\mathbf{x},\mathbf{y}) d(\mathbf{x},\mathbf{y}) - 3\beta I_1 + 3\beta^2 I_0 - a^2\beta^3\right). \end{aligned} \tag{55}$$

We are thus left with the computation of $I_3 = \int_{\Omega^2} \phi^3(\mathbf{x},\mathbf{y}) d(\mathbf{x},\mathbf{y})$ that we decompose as in section 2 (see Eq. (33) and (34)):

$$I_3 = \int_{\Omega^2} \phi^3(\mathbf{x},\mathbf{y}) d(\mathbf{x},\mathbf{y}) = (a - 2ur)\pi r^2 + \frac{u}{8}\int_0^2 \int_{A_h} \left(k(h,\mathbf{y}) + k(\mathbf{y},h)\right)^3 d\mathbf{y} dh, \tag{56}$$

where $k(h,\mathbf{y})$ and $k(\mathbf{y},h)$ are given by Eq. (35). A finite difference scheme with respect to variable h coupled with a Monte-Carlo sampling of $\mathbf{y}$ in A_h leads to the approximation

$$I_3 \approx a^2\beta\left(1 + 0.76\frac{ur}{a}\right). \tag{57}$$

Finally, using Supplementary Table **S1** in Eq. (55), we obtain

$$\mathbb{E}\left\{S_2\right\} \approx 4\tilde{\alpha}_2\left(\beta\left(1 + 0.76\frac{ur}{a}\right) - 3\beta^2\left(1 + 0.305\frac{ur}{a}\right)\right) \tag{58}$$

3.2.2 computation of $\mathbb{E}\left\{S_3\right\}$

Denoting

$$\mathbb{E}\left\{S_3^1\right\} = \alpha_3 \int_{\Omega^3} \phi_0^2(\mathbf{x},\mathbf{y})\phi_0(\mathbf{x},\mathbf{z}) d(\mathbf{x},\mathbf{y},\mathbf{z}) \tag{59}$$

and

$$\mathbb{E}\left\{S_3^2\right\} = \alpha_3 \int_{\Omega^3} \phi_0(\mathbf{x},\mathbf{y})\phi_0(\mathbf{x},\mathbf{z})\phi_0(\mathbf{y},\mathbf{z}) d(\mathbf{x},\mathbf{y},\mathbf{z}), \tag{60}$$

we have

$$\mathbb{E}\left\{S_3\right\} = 24\mathbb{E}\left\{S_3^1\right\} + 8\mathbb{E}\left\{S_3^2\right\}, \tag{61}$$

Expanding $\mathbb{E}\left\{S_3^1\right\}$ and using Monte-Carlo numerical integration to account for Ω boundaries we find that $\mathbb{E}\left\{S_3^1\right\} \approx 0$. We then expand $\mathbb{E}\left\{S_3^2\right\}$, as

$$\mathbb{E}\left\{S_3^2\right\} = \alpha_3\left(\int_{\Omega^3} \phi(\mathbf{x},\mathbf{y})\phi(\mathbf{x},\mathbf{z})\phi(\mathbf{y},\mathbf{z}) d(\mathbf{x},\mathbf{y},\mathbf{z}) - 3\beta I_2 + 3a\beta^2 I_0 - a^3\beta^3\right). \tag{62}$$

and are left with the computation of

$$I_4 = \int_{\Omega^3} \phi(\mathbf{x},\mathbf{y})\phi(\mathbf{x},\mathbf{z})\phi(\mathbf{y},\mathbf{z}) d(\mathbf{x},\mathbf{y},\mathbf{z}). \tag{63}$$

First, for $\mathbf{x}$ such that $|\mathbf{x} - \partial\Omega| > 2r$, there is no boundary correction and we decompose $I_4 = I_4^{in} + I_4^{border}$ where

$$\begin{aligned} I_4^{in} &= \int_{\Omega^3} \mathbf{1}_{\{|\mathbf{x}-\partial\Omega|>2r\}} \mathbf{1}_{\{|\mathbf{x}-\mathbf{y}|<r\}} \mathbf{1}_{\{|\mathbf{x}-\mathbf{z}|<r\}} \mathbf{1}_{\{|\mathbf{y}-\mathbf{z}|<r\}} d(\mathbf{x},\mathbf{y},\mathbf{z}) \\ &= (a - 2ur)\int_{\Omega^2} \mathbf{1}_{\{|\mathbf{x}-\mathbf{y}|<r\}} \mathbf{1}_{\{|\mathbf{x}-\mathbf{z}|<r\}} \mathbf{1}_{\{|\mathbf{y}-\mathbf{z}|<r\}} d(\mathbf{y},\mathbf{z}), \end{aligned} \tag{64}$$

and

$$\begin{aligned} I_4^{border} &= \frac{u}{8}\int_0^{2r}\int_{A_h} \left(k(h,\mathbf{y}) + k(\mathbf{y},h)\right) \\ &\quad \int_{A_h} \left(k(h,\mathbf{z}) + k(\mathbf{z},h)\right) \mathbf{1}_{\{|\mathbf{y}-\mathbf{z}|<r\}} \left(k(\mathbf{y},\mathbf{z}) + k(\mathbf{z},\mathbf{y})\right) d\mathbf{z} d\mathbf{y} dh. \end{aligned} \tag{65}$$

We then rewrite I_4^{in} as

$$I_4^{in} = (a - 2ur)\left(\pi r^2\right)^2 \Pr\left\{|\mathbf{y} - \mathbf{z}| < r \text{ given that } (\mathbf{y}, \mathbf{z}) \in b(\mathbf{x}, r)\right\}. \tag{66}$$

Because $\mathbf{y}$ and $\mathbf{z}$ are uniformly distributed in $b(\mathbf{x}, r)$, we have

$$\Pr\left\{|\mathbf{y} - \mathbf{z}| < r \text{ given that } (\mathbf{y}, \mathbf{z}) \in b(\mathbf{x}, r)\right\} = \frac{1}{\left(\pi r^2\right)^2} \int_{b(\mathbf{x},r)} |b(\mathbf{y}, r) \cap b(\mathbf{x}, r)| d\mathbf{y} \tag{67}$$

where $b(\mathbf{x}, r)$ and $b(\mathbf{y}, r)$ are the ball centered at $\mathbf{x}$ and $\mathbf{y}$ with radius r. Considering local polar coordinates: $\mathbf{y}(0 \leq r_{\mathbf{y}} \leq r, 0 \leq \theta_{\mathbf{y}} \leq 2\pi)$ around $\mathbf{x}(0, 0)$, we have

$$I_4^{in} = (a - 2ur) \int_{r_{\mathbf{y}}=0}^{r} \int_{\theta_{\mathbf{y}}=0}^{2\pi} A(r_{\mathbf{y}}, \theta_{\mathbf{y}}, r) r_{\mathbf{y}} d(r_{\mathbf{y}}, \theta_{\mathbf{y}}) \tag{68}$$

where $A(r_{\mathbf{y}}, \theta_{\mathbf{y}}, r) = |b(\mathbf{x}(0, 0), r) \cap b(\mathbf{y}(r_{\mathbf{y}}, \theta_{\mathbf{y}}), r)|$ is equal to [3]

$$A(r_{\mathbf{y}}, \theta_{\mathbf{y}}, r) = A(r_{\mathbf{y}}, r) = 2r^2 cos^{-1}\left(\frac{r_{\mathbf{y}}}{2r}\right) - \frac{r_{\mathbf{y}}}{2}\sqrt{4r^2 - r_{\mathbf{y}}^2}. \tag{69}$$

Finally, a direct integration I_4^{in} using Eq. (69) yields

$$I_4^{in} = (a - 2ur) 2\pi r^4 \left(\frac{\pi}{2} - \frac{3\sqrt{3}}{8}\right), \tag{70}$$

that is

$$I_4^{in} = (a - 2ur) a^2 \beta^2 \left(1 - \frac{3\sqrt{3}}{4\pi}\right) \approx (a - 2ur) a^2 \beta^2 0.587, \tag{71}$$

On the other hand, a numerical integration of I_4^{border} gives

$$I_4^{border} \approx a^3 \beta^2 1.38 \frac{ur}{a}, \tag{72}$$

leading to

$$I_4 = I_4^{in} + I_4^{border} \approx a^3 \beta^2 \left(0.587 + 0.207 \frac{ur}{a}\right). \tag{73}$$

Finally, reinjecting Eq. (73) in Eq. (62), and given that $\mathbb{E}\left\{S_3^1\right\} \approx 0$, we obtain

$$\mathbb{E}\left\{S_3\right\} \approx 8\mathbb{E}\left\{S_3^2\right\} = 8\tilde{\alpha}_3 \left(\beta^2 \left(0.587 + 0.207 \frac{ur}{a}\right) - \beta^3\right). \tag{74}$$

3.2.3 computation of $\mathbb{E}\{S_4\}$

We decompose the computation of $\mathbb{E}\{S_4\}$ as follows

$$\mathbb{E}\left\{S_4\right\} = a_4^1 \mathbb{E}\left\{S_4^1\right\} + a_4^2 \mathbb{E}\left\{S_4^2\right\} + a_4^3 \mathbb{E}\left\{S_4^3\right\} = 6\mathbb{E}\left\{S_4^1\right\} + 8\mathbb{E}\left\{S_4^2\right\} + 24\mathbb{E}\left\{S_4^3\right\} \tag{75}$$

with

$$\mathbb{E}\left\{S_4^1\right\} = \alpha_4 \int_{\Omega^4} \phi_0^2(\mathbf{x}, \mathbf{y}) \phi_0(\mathbf{z}, \mathbf{w}) d(\mathbf{x}, \mathbf{y}, \mathbf{z}, \mathbf{w}), \tag{76}$$

$$\mathbb{E}\left\{S_4^2\right\} = \alpha_4 \int_{\Omega^4} \phi_0(\mathbf{x}, \mathbf{y}) \phi_0(\mathbf{x}, \mathbf{z}) \phi_0(\mathbf{x}, \mathbf{w}) d(\mathbf{x}, \mathbf{y}, \mathbf{z}, \mathbf{w}), \tag{77}$$

and,

$$\mathbb{E}\left\{S_4^3\right\} = \alpha_4 \int_{\Omega^4} \phi_0(\mathbf{x}, \mathbf{y}) \phi_0(\mathbf{y}, \mathbf{z}) \phi_0(\mathbf{z}, \mathbf{w}) d(\mathbf{x}, \mathbf{y}, \mathbf{z}, \mathbf{w}). \tag{78}$$

First $\int_{\Omega^2} \phi_0(\mathbf{z},\mathbf{w})d(\mathbf{z},\mathbf{w}) = 0$ leads to $\mathbb{E}\left\{S_4^1\right\} = 0$. We then expand $\mathbb{E}\left\{S_4^2\right\}$ as

$$\mathbb{E}\left\{S_4^2\right\} = \alpha_4 \left(\int_{\Omega^4} \phi(\mathbf{x},\mathbf{y})\phi(\mathbf{x},\mathbf{z})\phi(\mathbf{x},\mathbf{w})d(\mathbf{x},\mathbf{y},\mathbf{z},\mathbf{w}) - 3\beta a I_2 + 3\beta^2 a^2 I_0 - a^4\beta^3 \right), \tag{79}$$

and decompose $\int_{\Omega^4} \phi(\mathbf{x},\mathbf{y})\phi(\mathbf{x},\mathbf{z})\phi(\mathbf{x},\mathbf{w})d(\mathbf{x},\mathbf{y},\mathbf{z},\mathbf{w})$ as

$$\begin{aligned} \int_{\Omega^4} \phi(\mathbf{x},\mathbf{y})\phi(\mathbf{x},\mathbf{z})\phi(\mathbf{x},\mathbf{w})d(\mathbf{x},\mathbf{y},\mathbf{z},\mathbf{w}) &= (a-2ur)a^3\beta^3 \\ &+ \frac{u}{8}\int_0^{2r} \left(\int_{A_h} (k(h,\mathbf{y}) + k(\mathbf{y},h))\, d\mathbf{y} \right)^3 dh. \end{aligned} \tag{80}$$

Numerical integration of Eq. (80) gives

$$\int_{\Omega^4} \phi(\mathbf{x},\mathbf{y})\phi(\mathbf{x},\mathbf{z})\phi(\mathbf{x},\mathbf{w})d(\mathbf{x},\mathbf{y},\mathbf{z},\mathbf{w}) \approx a^4\beta^3 \left(1 + 0.02\frac{ur}{a}\right). \tag{81}$$

and using Supplementary Table **S1** in Eq. (79), we compute that

$$\mathbb{E}\left\{S_4^2\right\} \approx 0. \tag{82}$$

Similarly, we expand $\mathbb{E}\left\{S_4^3\right\}$ as

$$\mathbb{E}\left\{S_4^3\right\} = \alpha_4 \left(\int_{\Omega^4} \phi(\mathbf{x},\mathbf{y})\phi(\mathbf{y},\mathbf{z})\phi(\mathbf{z},\mathbf{w})d(\mathbf{x},\mathbf{y},\mathbf{z},\mathbf{w}) - \beta\left(2aI_2 + I_0^2\right) + 2a^4\beta^3 \right) \tag{83}$$

and we are left with the numerical integration of $I_5 = \int_{\Omega^4} \phi(\mathbf{x},\mathbf{y})\phi(\mathbf{y},\mathbf{z})\phi(\mathbf{z},\mathbf{w})d(\mathbf{x},\mathbf{y},\mathbf{z},\mathbf{w})$. For $\mathbf{y}$ such that $|\mathbf{y} - \partial\Omega| > 3r$, points $\mathbf{x}$ and $\mathbf{z}$ such that $|\mathbf{x}-\mathbf{y}| < r$ and $|\mathbf{z}-\mathbf{y}| < r$ are at a minimal distance of $2r$ from the domain boundary $\partial\Omega$ and there is no boundary correction in I_5. We thus decompose I_5 as

$$\begin{aligned} I_5 = (a-3ur)\left(\pi r^2\right)^3 + \frac{u}{8}\int_0^{3r} \int_{A_h} (k(\mathbf{x},h) + k(h,\mathbf{x}))\, d\mathbf{x} \\ \int_{A_h} (k(\mathbf{z},h) + k(h,\mathbf{z})) \int_{A_\mathbf{z}} (k(\mathbf{z},\mathbf{w}) + k(\mathbf{w},\mathbf{z}))\, d\mathbf{w}d\mathbf{z}dh \end{aligned} \tag{84}$$

where $A_\mathbf{z} = b(\mathbf{z},r) \cap \Omega$. We then used a finite difference algorithm with respect to the variable h ($n_h = \frac{3r}{dh}$ steps of size $dh = 0.001$) coupled with a Monte-Carlo sampling of $\mathbf{x}$ and $\mathbf{z}$ in each $A_{h_j = j.dh}$ for $1 \le j \le n_h$ ($n_\mathbf{x} = n_\mathbf{z} = 1000$ random draws $\mathbf{x}_i, \mathbf{z}_p$, $1 \le i, p \le n$) as well as a sampling of $\mathbf{w}$ in each $A_{\mathbf{z}_p}$ related to each random draw $\mathbf{z}_p$ ($n_\mathbf{w} = 1000$ random draws $\mathbf{w}_k$, $1 \le k \le n_\mathbf{w}$) and approximate

$$\begin{aligned} &\int_0^{3r} \int_{A_h} (k(\mathbf{x},h) + k(h,\mathbf{x}))\, d\mathbf{x} \int_{A_h} (k(\mathbf{z},h) + k(h,\mathbf{z})) \int_{A_\mathbf{z}} (k(\mathbf{z},\mathbf{w}) + k(\mathbf{w},\mathbf{z}))\, d\mathbf{w}d\mathbf{z}dh \\ \approx\ & \sum_{j=1}^{n_h} \frac{A_{h_j}}{n_\mathbf{x}} \sum_{i}^{n_\mathbf{x}} (k(h_j,\mathbf{x}_i) + k(\mathbf{x}_i,h_j)) \\ & \frac{A_{h_j}}{n_\mathbf{z}} \sum_{p=1}^{n_\mathbf{z}} \left\{ (k(h_j,\mathbf{z}_p) + k(\mathbf{z}_p,h_j)) \frac{A_{\mathbf{z}_p}}{n_\mathbf{w}} \sum_{k=1}^{n_\mathbf{w}} (k(\mathbf{z}_p,\mathbf{w}_k) + k(\mathbf{w}_k,\mathbf{z}_p)) \right\} dh, \end{aligned} \tag{85}$$

leading to

$$I_5 \approx a^4\beta^3 \left(1 + 0.0153\frac{ur}{a}\right). \tag{86}$$

Finally, reinjecting Monte-Carlo approximation of I_5 in Eq. (83), we obtain

$$\mathbb{E}\left\{S_4^3\right\} \approx \tilde{\alpha}_4\beta^3 0.002\frac{ur}{a}, \tag{87}$$

leading to

$$\mathbb{E}\left\{S_4\right\} \approx 24\mathbb{E}\left\{S_4^3\right\} = 24\tilde{\alpha}_4\beta^3 0.002\frac{ur}{a},. \tag{88}$$

3.3 Conclusion

Finally, because $\int_{\Omega^2} \phi_0(\mathbf{x},\mathbf{y})d(\mathbf{x},\mathbf{y}) = 0$, we have

$$\mathbb{E}\{S_5\} = 12\alpha_5 \int_{\Omega^3} \phi_0(\mathbf{x},\mathbf{y})\phi_0(\mathbf{x},\mathbf{r})d(\mathbf{x},\mathbf{y},\mathbf{r}) \int_{\Omega^2} \phi_0(\mathbf{z},\mathbf{w})d(\mathbf{z},\mathbf{w}) = 0, \quad (89)$$

and

$$\mathbb{E}\{S_6\} = \alpha_6 \int_{\Omega^2} \phi_0(\mathbf{x},\mathbf{y})d(\mathbf{x},\mathbf{y}) \int_{\Omega^2} \phi_0(\mathbf{z},\mathbf{w})d(\mathbf{z},\mathbf{w}) \int_{\Omega^2} \phi_0(\mathbf{r},\mathbf{u})d(\mathbf{r},\mathbf{u}) = 0. \quad (90)$$

Consequently, using expressions of $\mathbb{E}\{S_2\}$ (Eq. (58)), $\mathbb{E}\{S_3\}$ (Eq. (74)) and $\mathbb{E}\{S_4\}$ (Eq. (88)) in Eq. (50) we obtain

$$\begin{aligned}\mathbb{E}\left\{(K(r,n)-\mathbb{E}\{K(r,n)\})^3\right\} &= \frac{a^3}{(n(n-1))^3}\sum_{j=2}^{j=4}\mathbb{E}\{S_j\}\\ = & \frac{4a^3}{(n(n-1))^2}\left[\beta\left(1+0.76\frac{ur}{a}\right)+\beta^2\left(\left(-3+1.173\frac{\tilde{\alpha}_3}{\tilde{\alpha}_2}\right)+\left(-0.915+\frac{\tilde{\alpha}_3}{\tilde{\alpha}_2}0.414\right)\frac{ur}{a}\right)\right.\\ + & \left.\beta^3\left(-2\frac{\tilde{\alpha}_3}{\tilde{\alpha}_2}+0.012\frac{\tilde{\alpha}_4}{\tilde{\alpha}_2}\frac{ur}{a}\right)\right], \end{aligned} \quad (91)$$

which simplifies for $n \gg 1$ to

$$\begin{aligned}&\mathbb{E}\left\{(K(r,n)-\mathbb{E}\{K(r,n)\})^3\right\}\\ = & \frac{4}{\lambda^3}\left(\frac{\beta}{n}\left(1+0.76\frac{ur}{a}\right)+\beta^2\left(1.173+0.414\frac{ur}{a}\right)+\beta^3\left(-2+0.012n\frac{ur}{a}\right)\right). \end{aligned} \quad (92)$$

4 computation of $\mathbb{E}\left\{(K(r,n)-\mathbb{E}\{K(r,n)\})^4\right\}$

We have

$$\mathbb{E}\left\{(K(r,n)-\mathbb{E}\{K(r,n)\})^4\right\} = \frac{a^4}{(n(n-1))^4}\mathbb{E}\left\{\left(\sum_{\mathbf{x}\neq\mathbf{y}}\phi_0(\mathbf{x},\mathbf{y})\right)^4\right\}, \quad (93)$$

and following the method of section 3, we expand $\mathbb{E}\left\{\left(\sum_{\mathbf{x}\neq\mathbf{y}}\phi_0(\mathbf{x},\mathbf{y})\right)^4\right\}$ as

$$\mathbb{E}\left\{\left(\sum_{\mathbf{x}\neq\mathbf{y}}\phi_0(\mathbf{x},\mathbf{y})\right)^4\right\} = \sum_{j=2}^{8}\mathbb{E}\left\{\tilde{S}_j\right\} \quad (94)$$

where $\tilde{S}_j$ is the sum of the terms containing j different points:

$$\tilde{S}_2 = \tilde{a}_2\sum_{\mathbf{x}\neq\mathbf{y}}\phi_0^4(\mathbf{x},\mathbf{y}), \quad (95)$$

$$\begin{aligned}\tilde{S}_3 &= a_3^1\sum_{\mathbf{x}\neq\mathbf{y}\neq\mathbf{z}}\phi_0^3(\mathbf{x},\mathbf{y})\phi_0(\mathbf{x},\mathbf{z}) + a_3^2\sum_{\mathbf{x}\neq\mathbf{y}\neq\mathbf{z}}\phi_0^2(\mathbf{x},\mathbf{y})\phi_0^2(\mathbf{x},\mathbf{z})\\ &+ a_3^3\sum_{\mathbf{x}\neq\mathbf{y}\neq\mathbf{z}}\phi_0^2(\mathbf{x},\mathbf{y})\phi_0(\mathbf{x},\mathbf{z})\phi_0(\mathbf{z},\mathbf{y}), \end{aligned} \quad (96)$$

$$\begin{aligned}\tilde{S}_4 &= \tilde{a}_4^1 \sum_{\mathbf{x}\neq\mathbf{y}\neq\mathbf{z}\neq\mathbf{w}} \phi_0^3(\mathbf{x},\mathbf{y})\phi_0(\mathbf{z},\mathbf{w}) + \tilde{a}_4^2 \sum_{\mathbf{x}\neq\mathbf{y}\neq\mathbf{z}\neq\mathbf{w}} \phi_0^2(\mathbf{x},\mathbf{y})\phi_0^2(\mathbf{z},\mathbf{w}) \\ &+ \tilde{a}_4^3 \sum_{\mathbf{x}\neq\mathbf{y}\neq\mathbf{z}\neq\mathbf{w}} \phi_0^2(\mathbf{x},\mathbf{y})\phi_0(\mathbf{x},\mathbf{z})\phi_0(\mathbf{x},\mathbf{w}) + \tilde{a}_4^4 \sum_{\mathbf{x}\neq\mathbf{y}\neq\mathbf{z}\neq\mathbf{w}} \phi_0^2(\mathbf{x},\mathbf{y})\phi_0(\mathbf{x},\mathbf{z})\phi_0(\mathbf{y},\mathbf{w}) \\ &+ \tilde{a}_4^5 \sum_{\mathbf{x}\neq\mathbf{y}\neq\mathbf{z}\neq\mathbf{w}} \phi_0^2(\mathbf{x},\mathbf{y})\phi_0(\mathbf{x},\mathbf{z})\phi_0(\mathbf{z},\mathbf{w}) + \tilde{a}_4^6 \sum_{\mathbf{x}\neq\mathbf{y}\neq\mathbf{z}\neq\mathbf{w}} \phi_0(\mathbf{x},\mathbf{y})\phi_0(\mathbf{y},\mathbf{z})\phi_0(\mathbf{z},\mathbf{w})\phi_0(\mathbf{x},\mathbf{w}) \\ &+ \tilde{a}_4^7 \sum_{\mathbf{x}\neq\mathbf{y}\neq\mathbf{z}\neq\mathbf{w}} \phi_0(\mathbf{x},\mathbf{y})\phi_0(\mathbf{x},\mathbf{z})\phi_0(\mathbf{x},\mathbf{w})\phi_0(\mathbf{y},\mathbf{z}), \end{aligned} \tag{97}$$

$$\begin{aligned}\tilde{S}_5 &= \tilde{a}_5^1 \sum_{\mathbf{x}\neq\mathbf{y}\neq\mathbf{z}\neq\mathbf{w}\neq\mathbf{r}} \phi_0^2(\mathbf{x},\mathbf{y})\phi_0(\mathbf{z},\mathbf{w})\phi_0(\mathbf{x},\mathbf{r}) + \tilde{a}_5^2 \sum_{\mathbf{x}\neq\mathbf{y}\neq\mathbf{z}\neq\mathbf{w}\neq\mathbf{r}} \phi_0^2(\mathbf{x},\mathbf{y})\phi_0(\mathbf{z},\mathbf{w})\phi_0(\mathbf{z},\mathbf{r}) \\ &+ \tilde{a}_5^3 \sum_{\mathbf{x}\neq\mathbf{y}\neq\mathbf{z}\neq\mathbf{w}\neq\mathbf{r}} \phi_0(\mathbf{x},\mathbf{y})\phi_0(\mathbf{x},\mathbf{z})\phi_0(\mathbf{x},\mathbf{w})\phi_0(\mathbf{x},\mathbf{r}) \\ &+ \tilde{a}_5^4 \sum_{\mathbf{x}\neq\mathbf{y}\neq\mathbf{z}\neq\mathbf{w}\neq\mathbf{r}} \phi_0(\mathbf{x},\mathbf{y})\phi_0(\mathbf{x},\mathbf{z})\phi_0(\mathbf{x},\mathbf{w})\phi_0(\mathbf{y},\mathbf{r}) \\ &+ \tilde{a}_5^5 \sum_{\mathbf{x}\neq\mathbf{y}\neq\mathbf{z}\neq\mathbf{w}\neq\mathbf{r}} \phi_0(\mathbf{x},\mathbf{y})\phi_0(\mathbf{x},\mathbf{z})\phi_0(\mathbf{y},\mathbf{z})\phi_0(\mathbf{w},\mathbf{r}) \\ &+ \tilde{a}_5^6 \sum_{\mathbf{x}\neq\mathbf{y}\neq\mathbf{z}\neq\mathbf{w}\neq\mathbf{r}} \phi_0(\mathbf{x},\mathbf{y})\phi_0(\mathbf{x},\mathbf{z})\phi_0(\mathbf{y},\mathbf{w})\phi_0(\mathbf{z},\mathbf{r}), \end{aligned} \tag{98}$$

$$\begin{aligned}\tilde{S}_6 &= \tilde{a}_6^1 \sum_{\mathbf{x}\neq\mathbf{y}\neq\mathbf{z}\neq\mathbf{w}\neq\mathbf{r}\neq\mathbf{u}} \phi_0^2(\mathbf{x},\mathbf{y})\phi_0(\mathbf{z},\mathbf{w})\phi_0(\mathbf{r},\mathbf{u}) \\ &+ \tilde{a}_6^2 \sum_{\mathbf{x}\neq\mathbf{y}\neq\mathbf{z}\neq\mathbf{w}\neq\mathbf{r}\neq\mathbf{u}} \phi_0(\mathbf{x},\mathbf{y})\phi_0(\mathbf{x},\mathbf{z})\phi_0(\mathbf{x},\mathbf{w})\phi_0(\mathbf{r},\mathbf{u}) \\ &+ \tilde{a}_6^3 \sum_{\mathbf{x}\neq\mathbf{y}\neq\mathbf{z}\neq\mathbf{w}\neq\mathbf{r}\neq\mathbf{u}} \phi_0(\mathbf{x},\mathbf{y})\phi_0(\mathbf{y},\mathbf{z})\phi_0(\mathbf{z},\mathbf{w})\phi_0(\mathbf{r},\mathbf{u}) \\ &+ \tilde{a}_6^4 \sum_{\mathbf{x}\neq\mathbf{y}\neq\mathbf{z}\neq\mathbf{w}\neq\mathbf{r}\neq\mathbf{u}} \phi_0(\mathbf{x},\mathbf{y})\phi_0(\mathbf{x},\mathbf{z})\phi_0(\mathbf{w},\mathbf{r})\phi_0(\mathbf{w},\mathbf{u}), \end{aligned} \tag{99}$$

$$\tilde{S}_7 = \tilde{a}_7 \sum_{\mathbf{x}\neq\mathbf{y}\neq\mathbf{z}\neq\mathbf{w}\neq\mathbf{r}\neq\mathbf{u}\neq\mathbf{s}} \phi_0(\mathbf{x},\mathbf{y})\phi_0(\mathbf{x},\mathbf{z})\phi_0(\mathbf{w},\mathbf{r})\phi_0(\mathbf{u},\mathbf{s}) \tag{100}$$

and

$$\tilde{S}_8 = \tilde{a}_8 \sum_{\mathbf{x}\neq\mathbf{y}\neq\mathbf{z}\neq\mathbf{w}\neq\mathbf{r}\neq\mathbf{u}\neq\mathbf{s}\neq\mathbf{r}} \phi_0(\mathbf{x},\mathbf{y})\phi_0(\mathbf{z},\mathbf{w})\phi_0(\mathbf{r},\mathbf{u})\phi_0(\mathbf{s},\mathbf{r}). \tag{101}$$

4.1 computation of coefficients $\tilde{a}_2, \tilde{a}_3^1, \ldots \tilde{a}_8$

Computing the number of terms in each sum of Eq. (94), we get that

$$(n(n-1))^4 = \tilde{a}_2\tilde{\alpha}_2 + \sum_{j=1}^{3} \tilde{a}_3^j\tilde{\alpha}_3 + \sum_{j=1}^{7} \tilde{a}_4^j\tilde{\alpha}_4 + \sum_{j=1}^{6} \tilde{a}_5^j\tilde{\alpha}_5 + \sum_{j=1}^{4} \tilde{a}_6^j\tilde{\alpha}_6 + \tilde{a}_7\tilde{\alpha}_7 + \tilde{a}_8\tilde{\alpha}_8, \tag{102}$$

where $\tilde{\alpha}_i = n(n-1)\ldots(n-i+1)$ is the number of ways to choose an ordered subset of i different points among n. Expanding Eq. (102) and identifying polynomial coefficients, we obtain that

$$\tilde{a}_2 = 8, \sum_{j=1}^{3} \tilde{a}_3^j = 208, \sum_{j=1}^{7} \tilde{a}_4^j = 652, \sum_{j=1}^{6} \tilde{a}_5^j = 576, \sum_{j=1}^{4} \tilde{a}_6^j = 188, \tilde{a}_7 = 24 \text{ and } \tilde{a}_8 = 1. \tag{103}$$

Using similar counting arguments as in sub-section 3.1, we further obtain that

$$\tilde{a}_3^1 = 64, \tilde{a}_3^2 = 48 \text{ and } \tilde{a}_3^3 = 96 \text{ which verify } \sum_{j=1}^{3} \tilde{a}_3^j = 208, \tag{104}$$

$$\tilde{a}_4^1 = 16, \tilde{a}_4^2 = 12, \tilde{a}_4^3 = \tilde{a}_4^4 = 96, \tilde{a}_4^5 = 192, \tilde{a}_4^6 = 48 \text{ and } \tilde{a}_4^7 = 192, \\ \text{which verify } \sum_{j=1}^{7} \tilde{a}_4^j = 652, \tag{105}$$

$$\tilde{a}_5^1 = 96, \tilde{a}_5^2 = 48, \tilde{a}_5^3 = 16, \tilde{a}_5^4 = 192, \tilde{a}_5^5 = 32 \text{ and } \tilde{a}_5^1 = 192, \\ \text{which verify } \sum_{j=1}^{6} \tilde{a}_5^j = 576, \tag{106}$$

and

$$\tilde{a}_6^1 = 12, \tilde{a}_6^2 = 32, \tilde{a}_6^3 = 96 \text{ and } \tilde{a}_6^4 = 48 \text{ which verify } \sum_{j=1}^{4} \tilde{a}_6^j = 188. \tag{107}$$

4.2 computation of $\mathbb{E}\left\{\tilde{S}_j\right\}$ for $2 \leq j \leq 8$

4.2.1 computation of $\mathbb{E}\left\{\tilde{S}_2\right\}$

Assuming a uniform distribution of the points inside Ω we have

$$\mathbb{E}\left\{\tilde{S}_2\right\} = 8\int_{\Omega^2} \phi_0^4(\mathbf{x},\mathbf{y})d\alpha_2(\mathbf{x},\mathbf{y}), \tag{108}$$

where $\alpha_2 = n(n-1)a^{-2}\mu_4$ (see Eq. (3)), that we expand as

$$\begin{aligned}\mathbb{E}\left\{\tilde{S}_2\right\} &= 8\alpha_2 \int_{\Omega^2} \left(\phi^4(\mathbf{x},\mathbf{y}) - 4\beta\phi^3(\mathbf{x},\mathbf{y}) + 6\beta^2\phi^2(\mathbf{x},\mathbf{y}) - 4\beta^3\phi(\mathbf{x},\mathbf{y}) + \beta^4\right) d(\mathbf{x},\mathbf{y}) \\ &= 8\alpha_2 \left(\int_{\Omega^2} \phi^4(\mathbf{x},\mathbf{y})d(\mathbf{x},\mathbf{y}) - 4\beta I_3 + 6\beta^2 I_1 - 4\beta^3 I_0 + a^2\beta^4\right).\end{aligned} \tag{109}$$

We are thus left with the computation of $\int_{\Omega^2} \phi^4(\mathbf{x},\mathbf{y})d(\mathbf{x},\mathbf{y})$ that we numerically evaluate to

$$\int_{\Omega^2} \phi^4(\mathbf{x},\mathbf{y})d(\mathbf{x},\mathbf{y}) \approx a^2\beta\left(1 + 1.44\frac{ur}{a}\right). \tag{110}$$

Finally, using Supplementary Table **S1** in Eq. (109), we obtain

$$\mathbb{E}\left\{\tilde{S}_2\right\} \approx 8\tilde{\alpha}_2\left(\beta\left(1 + 1.44\frac{ur}{a}\right) - 4\beta^2\left(1 + 0.76\frac{ur}{a}\right) + 6\beta^3\left(1 + 0.305\frac{ur}{a}\right)\right) \tag{111}$$

4.2.2 computation of $\mathbb{E}\left\{\tilde{S}_3\right\}$

Because $\tilde{a}_3^1 = 64$, $\tilde{a}_3^2 = 48$ and $\tilde{a}_3^3 = 96$, we can re-write $\mathbb{E}\left\{\tilde{S}_3\right\}$ as

$$\mathbb{E}\left\{\tilde{S}_3\right\} = 64\mathbb{E}\left\{\tilde{S}_3^1\right\} + 48\mathbb{E}\left\{\tilde{S}_3^2\right\} + 96\mathbb{E}\left\{\tilde{S}_3^2\right\}, \tag{112}$$

with

$$\mathbb{E}\left\{\tilde{S}_3^1\right\} = \alpha_3 \int_{\Omega^3} \phi_0^3(\mathbf{x},\mathbf{y})\phi_0(\mathbf{x},\mathbf{z})d(\mathbf{x},\mathbf{y},\mathbf{z}), \tag{113}$$

$$\mathbb{E}\left\{\tilde{S}_3^2\right\} = \alpha_3 \int_{\Omega^3} \phi_0^2(\mathbf{x},\mathbf{y})\phi_0^2(\mathbf{x},\mathbf{z})d(\mathbf{x},\mathbf{y},\mathbf{z}), \tag{114}$$

and

$$\mathbb{E}\left\{\tilde{S}_3^3\right\} = \alpha_3 \int_{\Omega^3} \phi_0^2(\mathbf{x},\mathbf{y})\phi_0(\mathbf{x},\mathbf{z})\phi_0(\mathbf{z},\mathbf{y})d(\mathbf{x},\mathbf{y},\mathbf{z}). \tag{115}$$

Accounting for Ω boundaries at leading order and using Monte-Carlo numerical approximations (see sections 2 and 3), we find that

$$\mathbb{E}\left\{\tilde{S}_3^1\right\} \approx 0, \tag{116}$$

$$\mathbb{E}\left\{\tilde{S}_3^2\right\} \approx \tilde{\alpha}_3 \left(\beta^2\left(1+0.7\frac{ur}{a}\right) - 2\beta^3\left(1+0.3\frac{ur}{a}\right)\right). \tag{117}$$

and

$$\mathbb{E}\left\{\tilde{S}_3^3\right\} \approx \tilde{\alpha}_3 \left(\beta^2\left(1-\frac{3\sqrt{3}}{4\pi}\right) + 0.47\frac{ur}{a} - \beta^3\left(2.17+0.72\frac{ur}{a}\right)\right), \tag{118}$$

leading to

$$\mathbb{E}\left\{\tilde{S}_3\right\} = \tilde{\alpha}_3 \left(\beta^2\left(104.3+78.7\frac{ur}{a}\right) - \beta^3\left(304.3+97.9\frac{ur}{a}\right)\right). \tag{119}$$

4.2.3 computation of $\mathbb{E}\left\{\tilde{S}_4\right\}$

We decompose the computation of $\tilde{S}_4$ as follows

$$\mathbb{E}\left\{\tilde{S}_4\right\} = \sum_{j=1}^{7} \tilde{a}_4^j \mathbb{E}\left\{\tilde{S}_4^j\right\} \tag{120}$$

where coefficients $\tilde{a}_4^j$ are given by Eq. (105) and,

$$\mathbb{E}\left\{\tilde{S}_4^1\right\} = \alpha_4 \int_{\Omega^4} \phi_0^3(\mathbf{x},\mathbf{y})\phi_0(\mathbf{z},\mathbf{w})d(\mathbf{x},\mathbf{y},\mathbf{z},\mathbf{w}), \tag{121}$$

$$\mathbb{E}\left\{\tilde{S}_4^2\right\} = \alpha_4 \int_{\Omega^4} \phi_0^2(\mathbf{x},\mathbf{y})\phi_0^2(\mathbf{z},\mathbf{w})d(\mathbf{x},\mathbf{y},\mathbf{z},\mathbf{w}), \tag{122}$$

$$\mathbb{E}\left\{\tilde{S}_4^3\right\} = \alpha_4 \int_{\Omega^4} \phi_0^2(\mathbf{x},\mathbf{y})\phi_0(\mathbf{x},\mathbf{z})\phi_0(\mathbf{x},\mathbf{w})d(\mathbf{x},\mathbf{y},\mathbf{z},\mathbf{w}), \tag{123}$$

$$\mathbb{E}\left\{\tilde{S}_4^4\right\} = \alpha_4 \int_{\Omega^4} \phi_0^2(\mathbf{x},\mathbf{y})\phi_0(\mathbf{x},\mathbf{z})\phi_0(\mathbf{y},\mathbf{w})d(\mathbf{x},\mathbf{y},\mathbf{z},\mathbf{w}), \tag{124}$$

$$\mathbb{E}\left\{\tilde{S}_4^5\right\} = \alpha_4 \int_{\Omega^4} \phi_0^2(\mathbf{x},\mathbf{y})\phi_0(\mathbf{x},\mathbf{z})\phi_0(\mathbf{z},\mathbf{w})d(\mathbf{x},\mathbf{y},\mathbf{z},\mathbf{w}), \tag{125}$$

$$\mathbb{E}\left\{\tilde{S}_4^6\right\} = \alpha_4 \int_{\Omega^4} \phi_0(\mathbf{x},\mathbf{y})\phi_0(\mathbf{y},\mathbf{z})\phi_0(\mathbf{z},\mathbf{w})\phi_0(\mathbf{x},\mathbf{w})d(\mathbf{x},\mathbf{y},\mathbf{z},\mathbf{w}), \tag{126}$$

and

$$\mathbb{E}\left\{\tilde{S}_4^7\right\} = \alpha_4 \int_{\Omega^4} \phi_0(\mathbf{x},\mathbf{y})\phi_0(\mathbf{x},\mathbf{z})\phi_0(\mathbf{x},\mathbf{w})\phi_0(\mathbf{y},\mathbf{z})d(\mathbf{x},\mathbf{y},\mathbf{z},\mathbf{w}). \tag{127}$$

First $\int_{\Omega^2}\phi_0(\mathbf{x},\mathbf{y})d(\mathbf{x},\mathbf{y}) = 0$ leading to $\mathbb{E}\left\{\tilde{S}_4^1\right\} = 0$. Then, we have $\mathbb{E}\left\{\tilde{S}_4^2\right\} = \alpha_4\left(I_1 - a^2\beta^2\right)^2$ leading to (see Supplementary Table **S1**),

$$\mathbb{E}\left\{\tilde{S}_4^2\right\} \approx \tilde{\alpha}_4\left(\beta\left(1+0.305\frac{ur}{a}\right)-\beta^2\right)^2. \quad (128)$$

Expansion and numerical integration near the boundary of $\mathbb{E}\left\{\tilde{S}_4^3\right\}$, $\mathbb{E}\left\{\tilde{S}_4^4\right\}$ and $\mathbb{E}\left\{\tilde{S}_4^5\right\}$ gives $\mathbb{E}\left\{\tilde{S}_4^3\right\} \approx \mathbb{E}\left\{\tilde{S}_4^4\right\} \approx \mathbb{E}\left\{\tilde{S}_4^5\right\} \approx 0$. Conversely, expansion of $\mathbb{E}\left\{\tilde{S}_4^6\right\}$ yields

$$\begin{aligned}\mathbb{E}\left\{\tilde{S}_4^6\right\} &\approx \alpha_4\{\int_{\Omega^4}\phi(\mathbf{x},\mathbf{y})\phi(\mathbf{y},\mathbf{z})\phi(\mathbf{z},\mathbf{w})\phi(\mathbf{x},\mathbf{w})d(\mathbf{x},\mathbf{y},\mathbf{z},\mathbf{w}) \\ &- 4\beta I_5 + 4\beta^2 aI_2 - a^4\beta^4\} \quad (129)\end{aligned}$$

and we are left with the computation of

$$I_6 = \int_{\Omega^4}\phi(\mathbf{x},\mathbf{y})\phi(\mathbf{y},\mathbf{z})\phi(\mathbf{z},\mathbf{w})\phi(\mathbf{x},\mathbf{w})d(\mathbf{x},\mathbf{y},\mathbf{z},\mathbf{w}). \quad (130)$$

First, for $\mathbf{x}$ such that $|\mathbf{x}-\partial\Omega| > 3r$, there is no boundary correction and we decompose $I_6 = I_6^{in} + I_6^{border}$ where

$$\begin{aligned}I_6^{in} &= \int_{\Omega^4}\mathbf{1}_{\{|\mathbf{x}-\partial\Omega|>3r\}}\mathbf{1}_{\{|\mathbf{x}-\mathbf{y}|<r\}}\mathbf{1}_{\{|\mathbf{x}-\mathbf{w}|<r\}}\left\{\mathbf{1}_{\{|\mathbf{y}-\mathbf{z}|<r\}}\mathbf{1}_{\{|\mathbf{w}-\mathbf{z}|<r\}}d\mathbf{z}\right\}d(\mathbf{x},\mathbf{y},\mathbf{w}) \\ &= (a-3ur)\int_{\Omega^3}\mathbf{1}_{\{|\mathbf{x}-\mathbf{y}|<r\}}\mathbf{1}_{\{|\mathbf{x}-\mathbf{w}|<r\}}\left\{\mathbf{1}_{\{|\mathbf{y}-\mathbf{z}|<r\}}\mathbf{1}_{\{|\mathbf{w}-\mathbf{z}|<r\}}d\mathbf{z}\right\}d\mathbf{w}d\mathbf{y}, \quad (131)\end{aligned}$$

and

$$\begin{aligned}I_6^{border} &= \frac{u}{16}\int_0^{3r}\int_{A_h}\int_{A_h}\left(k(h,\mathbf{y})+k(\mathbf{y},h)\right)\left(k(h,\mathbf{w})+k(\mathbf{w},h)\right) \\ &\left\{\mathbf{1}_{\{|\mathbf{y}-\mathbf{z}|<r\}}\mathbf{1}_{\{|\mathbf{w}-\mathbf{z}|<r\}}\left(k(\mathbf{y},\mathbf{z})+k(\mathbf{z},\mathbf{y})\right)\left(k(\mathbf{w},\mathbf{z})+k(\mathbf{z},\mathbf{w})\right)d\mathbf{z}\right\}d\mathbf{w}d\mathbf{y}dh. \quad (132)\end{aligned}$$

We then rewrite I_6^{in} as

$$I_6^{in} = (a-3ur)\left(\pi r^2\right)^2\Pr\left\{|\mathbf{y}-\mathbf{z}|<r \text{ and } |\mathbf{w}-\mathbf{z}|<r \text{ given that } (\mathbf{y},\mathbf{w})\in b(\mathbf{x},r)\right\}. \quad (133)$$

Denoting $d(\mathbf{y},\mathbf{w}) = |\mathbf{y}-\mathbf{w}|$, and $\mathbf{z}$ being uniformly distributed in Ω, we have

$$\begin{aligned}&\Pr\left\{|\mathbf{y}-\mathbf{z}|<r \text{ and } |\mathbf{w}-\mathbf{z}|<r \text{ given that } (\mathbf{y},\mathbf{w})\in b(\mathbf{x},r)\right\} \\ &= \frac{1}{(\pi r^2)^2}\int_{b(\mathbf{x},r)^2}|b(\mathbf{y},r)\cap b(\mathbf{w},r)|d\mathbf{y}d\mathbf{w} = \frac{1}{(\pi r^2)^2}\int_{b(\mathbf{x},r)^2}A(d(\mathbf{y},\mathbf{w}),r)d\mathbf{y}d\mathbf{w} \quad (134)\end{aligned}$$

where $A(d(\mathbf{y},\mathbf{w}),r)$ is given by Eq. (69)

$$A(d(\mathbf{y},\mathbf{w}),r) = 2r^2cos^{-1}\left(\frac{(d(\mathbf{y},\mathbf{w})}{2r}\right) - \frac{(d(\mathbf{y},\mathbf{w})}{2}\sqrt{4r^2-(d(\mathbf{y},\mathbf{w})^2}. \quad (135)$$

$\mathbf{y}$ and $\mathbf{w}$ are uniformly distributed in $b(\mathbf{x},r)$ and we can thus consider local polar coordinates: $\mathbf{y}(0\le r_{\mathbf{y}}\le r, 0\le\theta_{\mathbf{y}}\le 2\pi)$ and $\mathbf{w}(0\le r_{\mathbf{w}}\le r, 0\le\theta_{\mathbf{w}}\le 2\pi)$ around $\mathbf{x}(0,0)$ leading to

$$d(\mathbf{y},\mathbf{w}) = d(r_{\mathbf{y}},r_{\mathbf{w}},\Theta=\theta_{\mathbf{y}}-\theta_{\mathbf{w}}) = \sqrt{r_{\mathbf{y}}^2+r_{\mathbf{w}}^2-2r_{\mathbf{y}}r_{\mathbf{w}}cos(\Theta)}, \quad (136)$$

and re-write I_6^{in} as

$$I_6^{in} = (a-3ur)2\pi\int_{r_{\mathbf{y}}=0}^{r}\int_{r_{\mathbf{w}}=0}^{r}\int_{\Theta=0}^{2\pi}A\left(d\left(r_{\mathbf{y}},r_{\mathbf{w}},\Theta\right),t\right)r_{\mathbf{y}}r_{\mathbf{w}}d(r_{\mathbf{y}},r_{\mathbf{w}},\Theta). \quad (137)$$

Finally, a numerical integration of I_6^{in} with a finite differences scheme in $r_{\mathbf{y}}$, $r_{\mathbf{w}}$ and Θ gives

$$I_6^{in} \approx (a - 3ur)0.46(a\beta)^3. \tag{138}$$

Furthermore, we approximate mumerically I_6^{border} as

$$I_6^{border} \approx 3ur(a\beta)^3 0.53\frac{ur}{a}, \tag{139}$$

leading to

$$I_6 = I_6^{in} + I_6^{border} \approx a^4\beta^3\left(0.46 + 0.21\frac{ur}{a}\right). \tag{140}$$

and using Supplementary Table **S1** in Eq. (129) we obtain

$$\mathbb{E}\left\{\tilde{S}_4^6\right\} \approx \tilde{\alpha}_4\left(\beta^3\left(0.46 + 0.21\frac{ur}{a}\right) - \beta^4\right). \tag{141}$$

Finally, numerical integration of $\mathbb{E}\left\{\tilde{S}_4^7\right\}$ gives $\mathbb{E}\left\{\tilde{S}_4^7\right\} \approx 0$, and reinjecting Eq. (141) and Eq. (128) in Eq. (120), we obtain

$$\mathbb{E}\left\{\tilde{S}_4\right\} \approx \tilde{\alpha}_4\left(12\left(\beta\left(1 + 0.305\frac{ur}{a}\right) - \beta^2\right)^2 + 48\beta^3\left(0.46 + 0.21\frac{ur}{a}\right) - 48\beta^4\right), \tag{142}$$

that is

$$\mathbb{E}\left\{\tilde{S}_4\right\} \approx \tilde{\alpha}_4\left(\beta^2\left(12 + 7.32\frac{ur}{a} + 1.116\left(\frac{ur}{a}\right)^2\right) + \beta^3\left(-1.92 + 2.69\frac{ur}{a}\right) - 36\beta^4\right) \tag{143}$$

4.2.4 computation of $\mathbb{E}\left\{\tilde{S}_5\right\}$

We decompose the computation of $\mathbb{E}\left\{\tilde{S}_5\right\}$ as follows

$$\mathbb{E}\left\{\tilde{S}_5\right\} = \sum_{j=1}^{6}\tilde{a}_5^j\mathbb{E}\left\{\tilde{S}_5^j\right\} \tag{144}$$

where coefficients $\tilde{a}_5^j$ are given by Eq. (106) and,

$$\mathbb{E}\left\{\tilde{S}_5^1\right\} = \alpha_5\int_{\Omega^5}\phi_0^2(\mathbf{x},\mathbf{y})\phi_0(\mathbf{z},\mathbf{w})\phi_0(\mathbf{x},\mathbf{r})d(\mathbf{x},\mathbf{y},\mathbf{z},\mathbf{w},\mathbf{r}), \tag{145}$$

$$\mathbb{E}\left\{\tilde{S}_5^2\right\} = \alpha_5\int_{\Omega^5}\phi_0^2(\mathbf{x},\mathbf{y})\phi_0(\mathbf{z},\mathbf{w})\phi_0(\mathbf{z},\mathbf{r})d(\mathbf{x},\mathbf{y},\mathbf{z},\mathbf{w},\mathbf{r}), \tag{146}$$

$$\mathbb{E}\left\{\tilde{S}_5^3\right\} = \alpha_5\int_{\Omega^5}\phi_0(\mathbf{x},\mathbf{y})\phi_0(\mathbf{x},\mathbf{z})\phi_0(\mathbf{x},\mathbf{w})\phi_0(\mathbf{x},\mathbf{r})d(\mathbf{x},\mathbf{y},\mathbf{z},\mathbf{w},\mathbf{r}), \tag{147}$$

$$\mathbb{E}\left\{\tilde{S}_5^4\right\} = \alpha_5\int_{\Omega^5}\phi_0(\mathbf{x},\mathbf{y})\phi_0(\mathbf{x},\mathbf{z})\phi_0(\mathbf{x},\mathbf{w})\phi_0(\mathbf{y},\mathbf{r})d(\mathbf{x},\mathbf{y},\mathbf{z},\mathbf{w},\mathbf{r}), \tag{148}$$

$$\mathbb{E}\left\{\tilde{S}_5^5\right\} = \alpha_5\int_{\Omega^5}\phi_0(\mathbf{x},\mathbf{y})\phi_0(\mathbf{x},\mathbf{z})\phi_0(\mathbf{y},\mathbf{z})\phi_0(\mathbf{w},\mathbf{r})d(\mathbf{x},\mathbf{y},\mathbf{z},\mathbf{w},\mathbf{r}), \tag{149}$$

and

$$\mathbb{E}\left\{\tilde{S}_5^6\right\} = \alpha_5\int_{\Omega^5}\phi_0(\mathbf{x},\mathbf{y})\phi_0(\mathbf{x},\mathbf{z})\phi_0(\mathbf{y},\mathbf{w})\phi_0(\mathbf{z},\mathbf{r})d(\mathbf{x},\mathbf{y},\mathbf{z},\mathbf{w},\mathbf{r}). \tag{150}$$

First $\int_{\Omega^2} \phi_0(\mathbf{x},\mathbf{y})d(\mathbf{x},\mathbf{y}) = 0$ leads to $\mathbb{E}\left\{\tilde{S}_5^1\right\} = \mathbb{E}\left\{\tilde{S}_5^5\right\} = 0$. Then, we have

$$\mathbb{E}\left\{\tilde{S}_5^2\right\} = \alpha_5\left(I_1 - a^2\beta^2\right)\left(I_2 - a^3\beta^2\right) \tag{151}$$

that is, using Supplementary Table **S1**

$$\mathbb{E}\left\{\tilde{S}_5^2\right\} \approx \tilde{\alpha}_5\beta^3\left(1 + 0.305\frac{ur}{a}\right)0.0066\frac{ur}{a} \tag{152}$$

Finally, expansion and numerical integration near the boundary of $\mathbb{E}\left\{\tilde{S}_5^3\right\}$, $\mathbb{E}\left\{\tilde{S}_5^4\right\}$ and $\mathbb{E}\left\{\tilde{S}_5^6\right\}$ gives $\mathbb{E}\left\{\tilde{S}_5^3\right\} \approx \mathbb{E}\left\{\tilde{S}_5^4\right\} \approx \mathbb{E}\left\{\tilde{S}_4^6\right\} \approx 0.$ leading to

$$\mathbb{E}\left\{\tilde{S}_5\right\} \approx \tilde{a}_5^2\mathbb{E}\left\{\tilde{S}_5^2\right\} \approx 48\tilde{\alpha}_5\beta^3\left(1 + 0.305\frac{ur}{a}\right)0.0066\frac{ur}{a}, \tag{153}$$

that is

$$\mathbb{E}\left\{\tilde{S}_5\right\} \approx \tilde{\alpha}_5\beta^3\left(0.317\frac{ur}{a} + 0.0966\left(\frac{ur}{a}\right)^2\right). \tag{154}$$

4.2.5 computation of $\mathbb{E}\left\{\tilde{S}_6\right\}$

We decompose the computation of $\mathbb{E}\left\{\tilde{S}_6\right\}$ as follows

$$\mathbb{E}\left\{\tilde{S}_6\right\} = \sum_{j=1}^{4}\tilde{a}_6^j\mathbb{E}\left\{\tilde{S}_6^j\right\} \tag{155}$$

where coefficients $\tilde{a}_6^j$ are given by Eq. (107) and,

$$\mathbb{E}\left\{\tilde{S}_6^1\right\} = \alpha_6\int_{\Omega^6}\phi_0^2(\mathbf{x},\mathbf{y})\phi_0(\mathbf{z},\mathbf{w})\phi_0(\mathbf{r},\mathbf{u})d(\mathbf{x},\mathbf{y},\mathbf{z},\mathbf{w},\mathbf{r},\mathbf{u}), \tag{156}$$

$$\mathbb{E}\left\{\tilde{S}_6^2\right\} = \alpha_6\int_{\Omega^6}\phi_0(\mathbf{x},\mathbf{y})\phi_0(\mathbf{x},\mathbf{z})\phi_0(\mathbf{x},\mathbf{w})\phi_0(\mathbf{r},\mathbf{u})d(\mathbf{x},\mathbf{y},\mathbf{z},\mathbf{w},\mathbf{r},\mathbf{u}), \tag{157}$$

$$\mathbb{E}\left\{\tilde{S}_6^3\right\} = \alpha_6\int_{\Omega^6}\phi_0(\mathbf{x},\mathbf{y})\phi_0(\mathbf{y},\mathbf{z})\phi_0(\mathbf{z},\mathbf{w})\phi_0(\mathbf{r},\mathbf{u})d(\mathbf{x},\mathbf{y},\mathbf{z},\mathbf{w},\mathbf{r},\mathbf{u}), \tag{158}$$

and

$$\mathbb{E}\left\{\tilde{S}_6^4\right\} = \alpha_6\int_{\Omega^6}\phi_0(\mathbf{x},\mathbf{y})\phi_0(\mathbf{x},\mathbf{z})\phi_0(\mathbf{w},\mathbf{r})\phi_0(\mathbf{w},\mathbf{u})d(\mathbf{x},\mathbf{y},\mathbf{z},\mathbf{w},\mathbf{r},\mathbf{u}), \tag{159}$$

Because $\int_{\Omega^2}\phi_0(\mathbf{x},\mathbf{y})d(\mathbf{x},\mathbf{y}) = 0$, $\mathbb{E}\left\{\tilde{S}_6^1\right\} = \mathbb{E}\left\{\tilde{S}_6^2\right\} = \mathbb{E}\left\{\tilde{S}_6^3\right\} = 0$, and we are thus left with the computation of $\mathbb{E}\left\{\tilde{S}_6^4\right\}$ that reads

$$\begin{aligned}\mathbb{E}\left\{\tilde{S}_6^4\right\} &= \alpha_6\int_{\Omega^6}\phi_0(\mathbf{x},\mathbf{y})\phi_0(\mathbf{x},\mathbf{z})\phi_0(\mathbf{w},\mathbf{r})\phi_0(\mathbf{w},\mathbf{u})d(\mathbf{x},\mathbf{y},\mathbf{z},\mathbf{w},\mathbf{r},\mathbf{u}) \\ &= \alpha_6\left(I_2 - a^3\beta^2\right)^2 \approx \tilde{\alpha}_6\beta^4\left(0.0066\frac{ur}{a}\right)^2. \end{aligned} \tag{160}$$

and we have that

$$\mathbb{E}\left\{\tilde{S}_6\right\} = \tilde{a}_6^4\mathbb{E}\left\{\tilde{S}_6^4\right\} \approx \tilde{\alpha}_6\beta^4 0.0021\left(\frac{ur}{a}\right)^2. \tag{161}$$

4.2.6 Conclusion

Because $\int_{\Omega^2} \phi_0(\mathbf{x}, \mathbf{y}) d(\mathbf{x}, \mathbf{y}) = 0$, $\tilde{S}_7 = \tilde{S}_8 = 0$, and

$$\mathbb{E}\left\{(K(r,n) - \mathbb{E}\{K(r,n)\})^4\right\} = \frac{a^4}{(n(n-1))^4} \sum_{j=2}^{6} \mathbb{E}\left\{\tilde{S}_j\right\}. \quad (162)$$

Reinfecting expressions of $\mathbb{E}\left\{\tilde{S}_j\right\}$, for $2 \leq j \leq 6$ (Eq. (111), (119), (143), (154) and (161)), we have

$$\begin{aligned} \mathbb{E}\left\{(K(r,n) - \mathbb{E}\{K(r,n)\})^4\right\} &= \frac{a^4}{(n(n-1))^3} \left(\beta \left(8 + 11.52\frac{ur}{a}\right)\right. \quad (163) \\ + \quad & \beta^2 \left(\left(-32 + 104.3\frac{\tilde{\alpha}_3}{\tilde{\alpha}_2} + 12\frac{\tilde{\alpha}_4}{\tilde{\alpha}_2}\right) + \left(-24.32 + 78.7\frac{\tilde{\alpha}_3}{\tilde{\alpha}_2} + 7.32\frac{\tilde{\alpha}_4}{\tilde{\alpha}_2}\right)\frac{ur}{a}\right. \\ + \quad & \left. 1.116\frac{\tilde{\alpha}_4}{\tilde{\alpha}_2}\left(\frac{ur}{a}\right)^2\right) \\ + \quad & \beta^3 \left(\left(48 - 304.3\frac{\tilde{\alpha}_3}{\tilde{\alpha}_2} - 1.92\frac{\tilde{\alpha}_4}{\tilde{\alpha}_2}\right) + \left(14.784 - 97.9\frac{\tilde{\alpha}_3}{\tilde{\alpha}_2} + 2.69\frac{\tilde{\alpha}_4}{\tilde{\alpha}_2} + 0.317\frac{\tilde{\alpha}_5}{\tilde{\alpha}_2}\right)\frac{ur}{a}\right. \\ + \quad & \left. \frac{\tilde{\alpha}_5}{\tilde{\alpha}_2} 0.0966 \left(\frac{ur}{a}\right)^2\right) \\ + \quad & \left. \beta^4 \left(-36\frac{\tilde{\alpha}_4}{\tilde{\alpha}_2} + 0.0021\frac{\tilde{\alpha}_6}{\tilde{\alpha}_2}\left(\frac{ur}{a}\right)^2\right)\right), \end{aligned}$$

which simplifies for $n \gg 1$ to

$$\begin{aligned} \mathbb{E}\left\{(K(r,n) - \mathbb{E}\{K(r,n)\})^4\right\} &= \frac{1}{\lambda^4} \left(\frac{\beta}{n^2} \left(8 + 11.52\frac{ur}{a}\right)\right. \quad (164) \\ + \quad & \frac{\beta^2}{n} \left((104.3 + 12n) + (78.7 + 7.32n)\frac{ur}{a} + 1.116n\left(\frac{ur}{a}\right)^2\right) \\ + \quad & \frac{\beta^3}{n} \left((-304.3 - 1.92n) + \left(-97.9 + 2.69n + 0.317n^2\right)\frac{ur}{a} + n^2 0.0966\left(\frac{ur}{a}\right)^2\right) \\ + \quad & \left. \beta^4 \left(-36 + 0.0021n^2\left(\frac{ur}{a}\right)^2\right)\right). \end{aligned}$$

5 Computation of $\gamma\left(\tilde{K}_M(r)\right)$ and $\kappa\left(\tilde{K}_M(r)\right)$

We defined in the main manuscript the mean statistic

$$\tilde{K}_M(r) = \frac{1}{M} \sum_{j=1}^{M} \tilde{K}^j(r, n_j) \quad (165)$$

where $\tilde{K}^j(r, n_j)$ is the modified Ripley's K function that is evaluated on the jth field of view. Thus,

$$\gamma\left(\tilde{K}_M(r)\right) = \frac{\mathbb{E}\left\{\left(\frac{1}{M}\sum_{j=1}^{M}\left(\tilde{K}^j(r,n_j) - \pi r^2\right)\right)^3\right\}}{\left(\text{var}\left\{\frac{1}{M}\sum_{j=1}^{M}\tilde{K}^j(r,n_j)\right\}\right)^{\frac{3}{2}}}. \quad (166)$$

For $i \neq j$, $\tilde{K}^j$ is independent of $\tilde{K}^i$ and for all $1 \leq j \leq M$, $\text{var}\left\{\tilde{K}^j(r,n_j)\right\} = 1$, thus,

$$\text{var}\left\{\frac{1}{M}\sum_{j=1}^{M}\tilde{K}^j(r,n_j)\right\} = \frac{1}{M^2}\sum_{j=1}^{M}\text{var}\left\{\tilde{K}^j(r,n_j)\right\} = \frac{1}{M}, \quad (167)$$

and we rewrite

$$\gamma\left(\tilde{K}_M(r)\right) = \frac{1}{M^{\frac{3}{2}}}\mathbb{E}\left\{\left(\sum_{j=1}^{M}\left(\tilde{K}^j(r,n_j) - \pi r^2\right)\right)^3\right\} \tag{168}$$

Then, we decompose

$$\begin{aligned} \left(\sum_{j=1}^{M}\left(\tilde{K}^j(r,n_j) - \pi r^2\right)\right)^3 &= \sum_{j=1}^{M}\left(\tilde{K}^j(r,n_j) - \pi r^2\right)^3 \\ + \quad & 3\sum_{j\neq i}\left(\tilde{K}^j(r,n_j) - \pi r^2\right)^2\left(\tilde{K}^i(r,n_j) - \pi r^2\right) \\ + \quad & 6\sum_{j\neq i\neq k}\left(\tilde{K}^j(r,n_j) - \pi r^2\right)\left(\tilde{K}^i(r,n_j) - \pi r^2\right)\left(\tilde{K}^k(r,n_j) - \pi r^2\right), \end{aligned} \tag{169}$$

and because $\mathbb{E}\left\{\tilde{K}^j(r,n_j) - \pi r^2\right\} = 0$, we have

$$\mathbb{E}\left\{\left(\sum_{j=1}^{M}\left(\tilde{K}^j(r,n_j) - \pi r^2\right)\right)^3\right\} = \sum_{j=1}^{M}\mathbb{E}\left\{\left(\tilde{K}^j(r,n_j) - \pi r^2\right)^3\right\}, \tag{170}$$

that is

$$\mathbb{E}\left\{\left(\sum_{j=1}^{M}\left(\tilde{K}^j(r,n_j) - \pi r^2\right)\right)^3\right\} = \sum_{j=1}^{M}\gamma\left(\tilde{K}^j(r,n_j)\right), \tag{171}$$

which leads to

$$\gamma\left(\tilde{K}_M(r)\right) = \frac{1}{M^{\frac{3}{2}}}\sum_{j=1}^{M}\gamma\left(\tilde{K}^j(r,n_j)\right). \tag{172}$$

Similarly, we have

$$\kappa\left(\tilde{K}_M(r)\right) = \frac{1}{M^2}\mathbb{E}\left\{\left(\sum_{j=1}^{M}\left(\tilde{K}^j(r,n_j) - \pi r^2\right)\right)^4\right\}, \tag{173}$$

and we decompose

$$\begin{aligned} \left(\sum_{j=1}^{M}\left(\tilde{K}^j(r,n_j) - \pi r^2\right)\right)^4 &= \sum_{j=1}^{M}\left(\tilde{K}^j(r,n_j) - \pi r^2\right)^4 \\ + \quad & 4\sum_{j\neq i}\left(\tilde{K}^j(r,n_j) - \pi r^2\right)^3\left(\tilde{K}^i(r,n_j) - \pi r^2\right) \\ + \quad & 3\sum_{j\neq i}\left(\tilde{K}^j(r,n_j) - \pi r^2\right)^2\left(\tilde{K}^i(r,n_j) - \pi r^2\right)^2 \\ + \quad & 6\sum_{j\neq i\neq k}\left(\tilde{K}^j(r,n_j) - \pi r^2\right)^2\left(\tilde{K}^i(r,n_j) - \pi r^2\right)\left(\tilde{K}^k(r,n_j) - \pi r^2\right) \\ + \quad & 24\sum_{j\neq i\neq k\neq l}\left(\tilde{K}^j(r,n_j) - \pi r^2\right)\left(\tilde{K}^i(r,n_j) - \pi r^2\right)\left(\tilde{K}^k(r,n_j) - \pi r^2\right)\left(\tilde{K}^l(r,n_j) - \pi r^2\right) \end{aligned} \tag{174}$$

which leads to

$$\mathbb{E}\left\{\left(\sum_{j=1}^{M}\left(\tilde{K}^j(r,n_j)-\pi r^2\right)\right)^4\right\} = \sum_{j=1}^{M}\mathbb{E}\left\{\left(\tilde{K}^j(r,n_j)-\pi r^2\right)^4\right\}$$
$$+ \quad 3\sum_{j\neq i}\mathbb{E}\left\{\left(\tilde{K}^j(r,n_j)-\pi r^2\right)^2\right\}\mathbb{E}\left\{\left(\tilde{K}^i(r,n_i)-\pi r^2\right)^2\right\}, \tag{175}$$

that is

$$\mathbb{E}\left\{\left(\sum_{j=1}^{M}\left(\tilde{K}^j(r,n_j)-\pi r^2\right)\right)^4\right\} = \sum_{j=1}^{M}\kappa\left(\tilde{K}^j(r,n_j)\right) + 3M\left(M-1\right). \tag{176}$$

Reinjecting Eq. 176 in Eq. 173, we obtain

$$\kappa\left(\tilde{K}_M(r)\right) = \frac{1}{M^2}\sum_{j=1}^{M}\kappa\left(\tilde{K}^j(r,n_j)\right) + 3\frac{M-1}{M}. \tag{177}$$

Table **S1**: Numerical approximations of major integrals

Denotation	Formula	Numerical approximation
I_0	$\int_{\Omega^2} \phi(\mathbf{x},\mathbf{y})d(\mathbf{x},\mathbf{y})$	$a^2\beta$
I_1	$\int_{\Omega^2} \phi(\mathbf{x},\mathbf{y})^2 d(\mathbf{x},\mathbf{y})$	$a^2\beta\left(1+0.305\frac{ur}{a}\right)$
I_2	$\int_{\Omega^3} \phi(\mathbf{x},\mathbf{y})\phi(\mathbf{x},\mathbf{z})d(\mathbf{x},\mathbf{y},\mathbf{z})$	$a^3\beta^2\left(1+0.0066\frac{ur}{a}\right)$
I_3	$\int_{\Omega^2} \phi^3(\mathbf{x},\mathbf{y})d(\mathbf{x},\mathbf{y})$	$a^2\beta\left(1+0.76\frac{ur}{a}\right)$
I_4	$\int_{\Omega^3} \phi(\mathbf{x},\mathbf{y})\phi(\mathbf{x},\mathbf{z})\phi(\mathbf{y},\mathbf{z})d(\mathbf{x},\mathbf{y},\mathbf{z})$	$a^3\beta^2\left(1-\frac{3\sqrt{3}}{4\pi}+0.207\frac{ur}{a}\right)$
I_5	$\int_{\Omega^4} \phi(\mathbf{x},\mathbf{y})\phi(\mathbf{y},\mathbf{z})\phi(\mathbf{z},\mathbf{w})d(\mathbf{x},\mathbf{y},\mathbf{z},\mathbf{w})$	$a^4\beta^3\left(1+1.0051\frac{ur}{a}\right)$
I_6	$\int_{\Omega^4} \phi(\mathbf{x},\mathbf{y})\phi(\mathbf{y},\mathbf{z})\phi(\mathbf{z},\mathbf{w})\phi(\mathbf{x},\mathbf{w})d(\mathbf{x},\mathbf{y},\mathbf{z},\mathbf{w})$	$a^4\beta^3\left(0.46+0.21\frac{ur}{a}\right)$

References

[1] Ripley B (1988) Statistical inference for spatial processes. Cambridge University Press.

[2] Getis A, Franklin J (1987) Second-order neighborhood analysis of mapped point patterns. Ecology 68: 473-477.

[3] Weisstein E. URL `http://mathworld.wolfram.com/Circle-CircleIntersection.html`.

[4] de Chaumont F, Dallongeville S, Chenouard N, Hervé N, Pop S, et al. (2012) Icy: an open bioimage informatics platform for extended reproducible research. Nat Methods 9: 690-6.

[5] Olivo-Marin JC (2002) Extraction of spots in biological images using multiscale products. Pattern Recognition 35: 1989-1996.
